# LRH-1 drives colon cancer cell growth by repressing the expression of the *CDKN1A* gene in a p53-dependent manner

**DOI:** 10.1093/nar/gkv948

**Published:** 2015-09-22

**Authors:** Holly B. Kramer, Chun-Fui Lai, Hetal Patel, Manikandan Periyasamy, Meng-Lay Lin, Stephan M. Feller, Frances V. Fuller-Pace, David W. Meek, Simak Ali, Laki Buluwela

**Affiliations:** 1 Department of Surgery & Cancer, Imperial College London, Hammersmith Hospital Campus, Du Cane Road, London W12 0NN, UK; 2 Institute of Molecular Medicine, Martin-Luther-University Halle-Wittenberg, Heinrich-Damerow-Str. 1, D-06120 Halle (Saale), Germany; 3 Division of Cancer Research, University of Dundee, Ninewells Hospital & Medical School, Dundee DD1 9SY, UK

## Abstract

Liver receptor homologue 1 (LRH-1) is an orphan nuclear receptor that has been implicated in the progression of breast, pancreatic and colorectal cancer (CRC). To determine mechanisms underlying growth promotion by LRH-1 in CRC, we undertook global expression profiling following siRNA-mediated LRH-1 knockdown in HCT116 cells, which require LRH-1 for growth and in HT29 cells, in which LRH-1 does not regulate growth. Interestingly, expression of the cell cycle inhibitor p21 (*CDKN1A*) was regulated by LRH-1 in HCT116 cells. p21 regulation was not observed in HT29 cells, where p53 is mutated. p53 dependence for the regulation of p21 by LRH-1 was confirmed by p53 knockdown with siRNA, while LRH-1-regulation of p21 was not evident in HCT116 cells where p53 had been deleted. We demonstrate that LRH-1-mediated p21 regulation in HCT116 cells does not involve altered p53 protein or phosphorylation, and we show that LRH-1 inhibits p53 recruitment to the p21 promoter, likely through a mechanism involving chromatin remodelling. Our study suggests an important role for LRH-1 in the growth of CRC cells that retain wild-type p53.

## INTRODUCTION

Colorectal cancer (CRC) is one of the most commonly diagnosed cancers worldwide (1). Although five-year survival rates over the last 40 years have greatly improved in many countries, CRC remains the second most common global cause of cancer deaths. CRC development occurs sequentially through the accumulation of genetic changes or mutations, resulting in unrestrained cellular proliferation and survival (2,3). The genetic changes leading to CRC are well defined, with aberrant activation of the Wnt/β-catenin signalling pathway and loss of the tumour suppressor p53 being especially important.

Liver receptor homologue 1 (LRH-1; also known as NR5A2) is a member of the NR5A, or Ftz-F1, sub-family of nuclear receptors (NRs) (4). LRH-1 is expressed in tissues of endodermal origin, including the intestine, pancreas, liver, ovaries and testes, as well as the adrenal gland and placenta (4–9). The Ftz-F1 sub-family feature a region known as the Ftz-F1 box, a 26aa motif located C-terminal to the DNA binding domain (DBD). NRs containing this region are able to bind to a 9 bp DNA consensus sequence, 5′-YCAAGGYCR-3′ (where Y is any pyrimidine and R is any purine) as monomers (4,10). While there is evidence that LRH-1 activity is regulated by binding of phospholipids in the ligand binding domain (LBD) (11–14), it is generally classed as an orphan NR. The LRH-1 LBD is held in a permanently active conformation (15), and its activity is primarily regulated by transcriptional co-activators, such as peroxisome proliferator-activated receptor γ coactivator-1α (PGC-1α), and co-repressors, such as the atypical NR small heterodimer partner (SHP; also known as NR0B2) (16,17). Homozygous deletion of LRH-1 is embryonic lethal at day E6.5 in mice, demonstrating its importance in development (18,19), and LRH-1 regulates the expression of octamer binding transcription factor 4 (Oct4), a transcription factor required for the maintenance of pluripotency in embryonic stem cells (18). Strikingly, LRH-1 can substitute for Oct4 in the reprogramming of murine somatic cells to induced pluripotent stem cells through regulation of Nanog, an important factor for maintaining pluripotency (20). Roles for LRH-1 have also been established in the regulation of bile acid and cholesterol homeostasis in the liver (4,21), in steroid hormone biosynthesis in the ovary (22), and in the maintenance of pregnancy (23).

LRH-1 has also been implicated in cancer development. Genome wide association studies have shown a link between LRH-1 and pancreatic cancer (24), and down-regulation of LRH-1 in pancreatic cancer cell lines inhibits proliferation, thought to be due to reduced expression of c-Myc and the cyclins D1 and E1 (25). LRH-1 expression is frequently detected in breast tumours and surrounding adipose tissue, and expression of LRH-1 with estrogen receptor α (ERα) is positively associated in human breast carcinoma tissues and in breast cancer cell lines (26–29). In breast cancer associated stroma, LRH-1 regulates expression of the cytochrome P450 aromatase (*CYP19*) enzyme by binding to promoter II of the *CYP19* gene, thereby contributing to the local biosynthesis of estrogen from 19 carbon steroids (30,31). In breast cancer cells, LRH-1 expression is induced by estrogen, via ERα, and LRH-1 regulates breast cancer cell growth (26,28). Regulation of growth involves direct modulation of ERα expression (28), stimulation of ERα recruitment to DNA, possibly by promoting co-factor recruitment and remodelling of chromatin to a more open state (32), and LRH-1 recruitment to regulatory regions of genes that enhance cell growth (33). LRH-1 also promotes breast cancer cell motility and invasion (34).

In the colon, LRH-1 has been implicated in intestinal tumour formation. Mice heterozygous for an adenomatous polyposis coli (APC) mutation and a LRH-1 inactivating mutation developed fewer intestinal tumours than mice harbouring the APC mutation only, and LRH-1 heterozygous mice developed fewer azoxymethane-induced aberrant crypt foci (35). LRH-1 is highly expressed in the intestinal crypts. In the crypts of mice heterozygous for LRH-1, reduced expression of cyclins D1 and E1, as well as reduced DNA synthesis, has been described. Promotion of the proliferation of intestinal cells by LRH-1 required synergism with β-catenin on the cyclin E1 and D1 gene promoters (36). In CRC cells, LRH-1 also regulates the expression of Cyp11A1 and Cyp11B1, steroidogenic enzymes that play a key role in regulating levels of immunomodulatory glucocorticoids, which act to suppress host immune responses (37).

To further investigate the mechanisms of LRH-1 action in CRC, we undertook gene expression microarray profiling in two CRC cell lines following siRNA-mediated LRH-1 knockdown to define the LRH-1 transcriptome. Pathway analysis of differentially regulated genes identified an important role for LRH-1 in the regulation of the cell cycle inhibitor p21. Interestingly, regulation of p21 by LRH-1 was dependent on p53 and was not observed if the p53 gene was mutated or deleted. Collectively, this work demonstrates a novel role for LRH-1 in the regulation of p21 levels in CRC that retain wild-type p53, and identifies LRH-1 as a potential target for the treatment of these tumours.

## MATERIALS AND METHODS

### Cell culture

Cell lines were obtained from the American Tissue Type Culture Collection and were maintained in the recommended culture media. HCT116 p53^−/−^ cells were kindly provide by Dr. B. Vogelstein (38). HCT116 and HCT116 p53^−/−^ cells were maintained in McCoy's 5A medium. HT29, LOVO and HCA46 cells were cultured in DMEM. H1299 cells were maintained in RPMI-1640 medium. All media were supplemented with 10% FCS.

### Plasmids

The Renilla luciferase reporter gene plasmid was pRL-CMV (Promega, UK). The p21 promoter firefly luciferase reporter plasmid, p21-Luc has been described (39), as has the p53 plasmid (40). HA-tagged LRH-1 (pCI-HA-LRH-1) was generated from pCI-LRH-1 (13), as described (32). pCI-HA-LRH-1 G95W was generated by site-directed mutagenesis using the Quickchange kit (Stratagene, UK), using oligonucleotides having the sequence 5′-CCGTGTGTGGAGATAAAGTGTCTTGGTACCATTATGG-3′.

### Reporter gene assays

H1299 cells, seeded in 24-well plates, were transfected with 100 ng of p21-luc, 1 ng p53, 1–100 ng LRH-1 and 10 ng of the renilla luciferase plasmid, pRL-CMV, using FuGENE HD (Promega). Luciferase activities were determined after 24 h, using the Dual-Glo Luciferase Assay kit (Promega). To control for transfection efficiency, firefly luciferase activities were calculated relative to Renilla luciferase activities.

### siRNA transfections

Cells were transfected with double-stranded RNA oligonucleotides to a final concentration of 5nM, using Lipofectamine^TM^ RNAiMAX (Invitrogen, UK) and the reverse transfection method, according to manufacturer's instructions. ON-TARGETPlus siRNAs for LRH-1 (Dharmacon, UK) have the sequences: 5′-AGAGAAAUUUGGACAGCUA-3′ (#1) and 5′-GGAGUGAGCUCUUAAUCCU-3′ (#2). Silencer Select siRNAs for TP53 (Ambion, UK) have the sequences: 5′-GUA AUC UAC UGG GAC GGA ATT-3′ (#1) and 5′-GAA AUU UGC GUG UGG AGU ATT-3′ (#2). siLUC control (P-002099–01–20; Dharmacon) was used as a negative control.

### Cell proliferation assays

Cell growth was determined using the sulphorhodamine B assay (SRB) (41). siRNA-transfected cells were seeded at a density of 3 × 10^3^ cells/well in 96-well plates. On the day of measurement, cells were fixed by the addition of 100 μl ice-cold 40% trichloroacetic acid (TCA), followed by incubation at 4°C for 1 h. Cells were washed in ddH_2_O and stained with 100 μl 0.4% SRB dye in 1% acetic acid for 1 h. Cells were washed five times in 1% acetic acid and air dried. Bound dye was solubilized by addition of 100 μl of 10 mM Tris-base. Absorbance was read at 492 nm.

### Real-time quantitative polymerase chain reaction

RNA was collected using RNeasy Mini Preparation Kit (QIAGEN, UK) according to the manufacturer's instructions. cDNA was synthesized from 2 μg RNA using RevertAid^TM^ M-MuLV reverse transcriptase (Fermentas, UK). Obtained cDNA was diluted 1:10 and 2 μl was used in each PCR. Gene expression analyses were carried out using an Applied Biosystems 7900HT Fast Real-Time PCR System with TaqMan^®^ gene expression assays (Applied Biosystems, UK) for LRH-1 (Hs00892377_m1), CDKN1A (Hs00355782_m1), TP53 (Hs01034249_m1) and GAPDH (Hs99999905_m1).

### Electrophoretic mobility shift assay (EMSA)

*In vitro* transcription of LRH-1 and LRH-1 G95W expression plasmids was performed with the T7 RNA polymerase kit (Invitrogen). RNA was *in vitro* translated using the Rabbit Reticulocyte Lysate System (Promega), according to manufacturer's instructions. EMSA was carried out as described previously (32).

### Western blotting

Whole cell lysates were prepared in radioimmune precipitation (RIPA) buffer (Sigma-Aldrich), supplemented with protease and phosphatase inhibitor cocktail tablets (Roche, UK), and protein concentration was determined using the Pierce BCA Protein Assay Kit (Thermo Fisher Scientific, UK). Western blotting was performed using 20 μg of protein lysate, as previously described (42). Antibodies for LRH-1 (ab41901), Bax (ab32503), p53-phospho-ser15 (ab1431), Mdm2 (ab16895) and β-actin (ab6276) were purchased from Abcam, UK. p21 (sc-397) and p53 (sc-126) were from Santa Cruz Biotechnologies, USA. p53-phospho-ser33 (#2528), p53-phospho-ser392 (#9281) were purchased from Cell Signaling, UK.

### Co-immunoprecipitation (Co-IP)

Co-IPs were carried out using the ImmunoCruz™ IP/WB Optima E System (Santa Cruz, UK), according to the manufacturer's instructions, with 1 mg whole cell lysate prepared by RIPA extraction for each IP. 2 μg of LRH-1 antibody or normal mouse IgG (Invitrogen) was used per IP. Following immunoprecipitation, samples were heated at 100°C for 3 min and 10 μl of immunoprecipitated complex was used for Western blotting. HRP conjugated ImmunoCruz™ E antibody was used as the secondary antibody.

### Chromatin immunoprecipitation (ChIP)

For transient transfections, HCT116 cells seeded in 15 cm dishes for 24 h were transfected with 5 μg pcDNA vector or pCI-HA-LRH-1 and 10 μg BSM carrier DNA using FuGENE HD. Lysates were prepared after 48 h. For siLRH-1 ChIP analysis, cells were transfected with LRH-1 siRNA and lysates were prepared 24 h after transfection. Where appropriate, cells were treated with DMSO or 10 μM etoposide for 24 h. ChIP was performed as previously described (28,32). ChIP primers have also previously been described (28,32,43). Antibodies for H3 (ab1791), H3K9Ac (ab4441), H3K27Ac (ab4729), H3K4me3 (ab8580) and H3K9me3 (ab8898) were purchased from Abcam, UK.

### Gene expression microarray analysis

HCT116 and HT29 cells were transfected with LRH-1 siRNA #1, #2 or with siLUC. Total RNA was prepared 24 h later using the RNeasy Mini Preparation Kit (QIAGEN). Following assessment of RNA integrity, four independent biological replicates for each siRNA treatment were used for microarray analysis. The analysis was performed using HumanHT-12 v4 Expression BeadChip (Illumina, UK). The BeadChip image data were pre-processed using GenomeStudio (Illumina). Raw data were filtered prior to normalization to remove non-expressed probes (if for a given probe the detection *P*-values for all samples were >0.05 it was considered not expressed). The expression data were log_2_ transformed and quantile-normalized using Partek Genomics Suite (Partek, USA). The microarray data have been deposited with the NCBI Gene Expression Omnibus (GEO) (http://ncbi.nlm.nih.gov/geo/) under accession number GSE65815. Pathway analyses were performed using the database for annotation, visualization and integrated discovery (DAVID; http://david.abcc.ncifcrf.gov) (44,45).

## RESULTS

### Differential regulation of CRC cell growth by LRH-1

**Figure 1.**
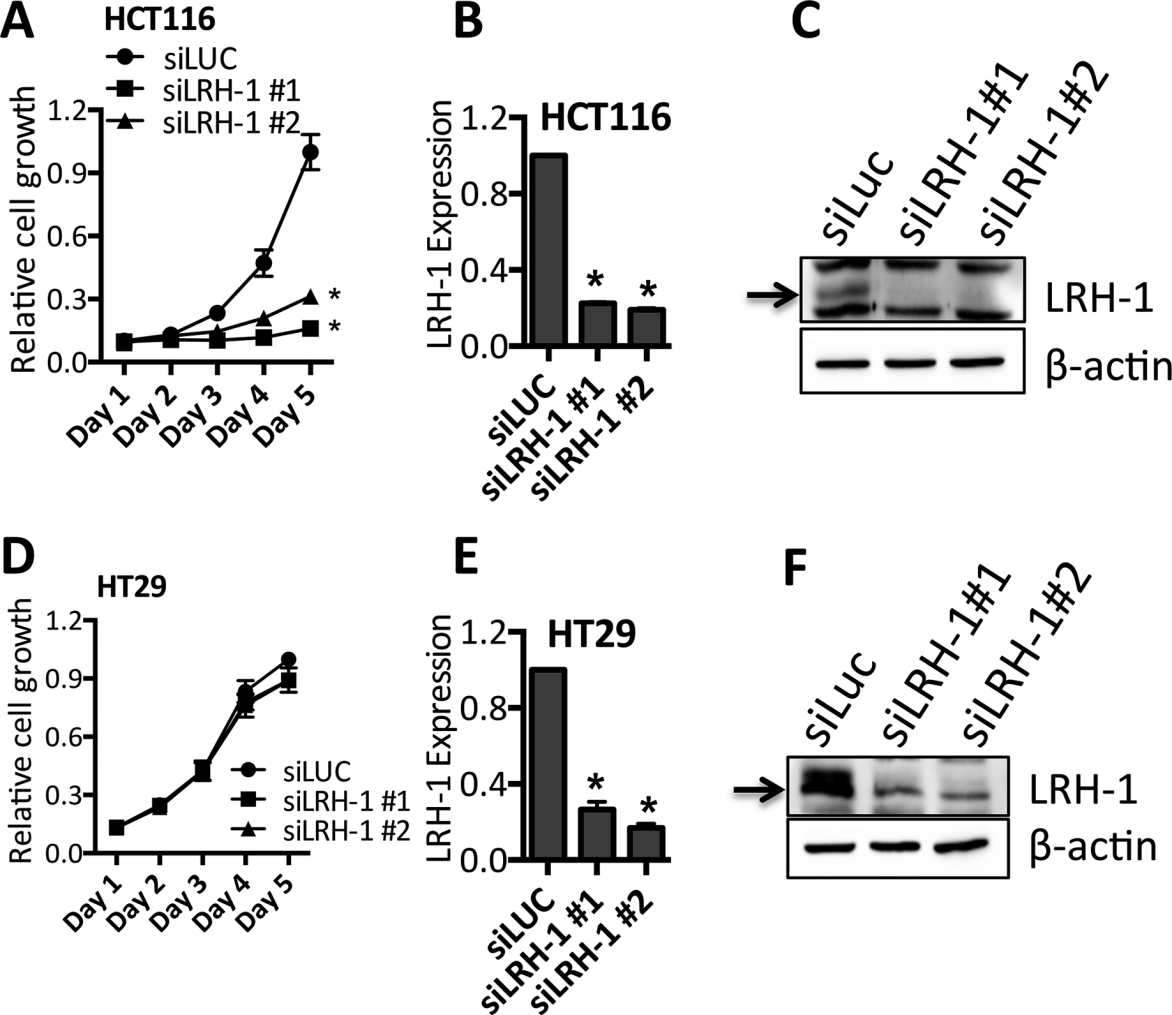
LRH-1 can regulate the growth of CRC cells. HCT116 and HT29 cells were transfected with two LRH-1 siRNAs. The non-targeting control siRNA (siLUC) served as a control for transfection. (**A**, **D**) The growth of cells transfected with siRNA was measured by SRB assay. (**B**, **E**) RNA prepared 72 h post-transfection was used in qRT-PCR for LRH-1. LRH-1 expression was normalised to GAPDH levels and is shown relative to expression in siLUC transfected controls. (**C**, **F**) Immunoblotting for LRH-1 and β-actin was carried out using lysates prepared 72 h following siRNA transfection. Graphical data show the means of three independent experiments, with error bars representing the SEM; * = *P* < 0.001.

Given the described role of LRH-1 in CRC, we investigated the importance of LRH-1 for CRC cell growth, following siRNA-mediated knockdown in two commonly used CRC cell lines, HCT116 and HT29. These lines were chosen since they show moderate or high LRH-1 expression (Supplementary Figure S1). Transfection with two independent LRH-1 siRNAs in HCT116 resulted in significant reduction in LRH-1 at both the mRNA and protein levels, which was accompanied by 85% (siLRH-1 #1) and 69% (siLRH-1 #2) growth inhibition relative to the control siRNA (siLUC) (Figure 1A–C). Interestingly, these siRNAs failed to elicit a substantial growth effect in HT29 cells (Figure 1D–F). It should be noted that several LRH-1 isoforms arising from alternative 5′ exon usage and/or alternative splicing yield LRH-1 polypeptides that differ in molecular mass by 5–10 kDa (28). Immunoblotting shows a 55 kDa LRH-1 polypeptide present in all CRC cell lines, expected to correspond to the 495aa LRH-1 variant. The larger 541aa variant 1 was absent in HCT116 cells.

### Gene expression microarray analysis for the identification of LRH-1 regulated genes in CRC cells

These results demonstrate a potential role for LRH-1 in the regulation of HCT116 growth, but do not provide a function for LRH-1 in HT29 cells. To identify LRH-1-regulated genes and to determine the possible mechanisms underlying the differential role of LRH-1 on growth in these cell lines, global gene expression profiling was undertaken following LRH-1 knockdown in both cell lines, using the Illumina HumanHT-12 v4 Expression BeadChip. Four independent RNA samples for each of the two LRH-1 siRNAs were analysed, to facilitate robust statistical analysis of the microarray data.

**Figure 2.**
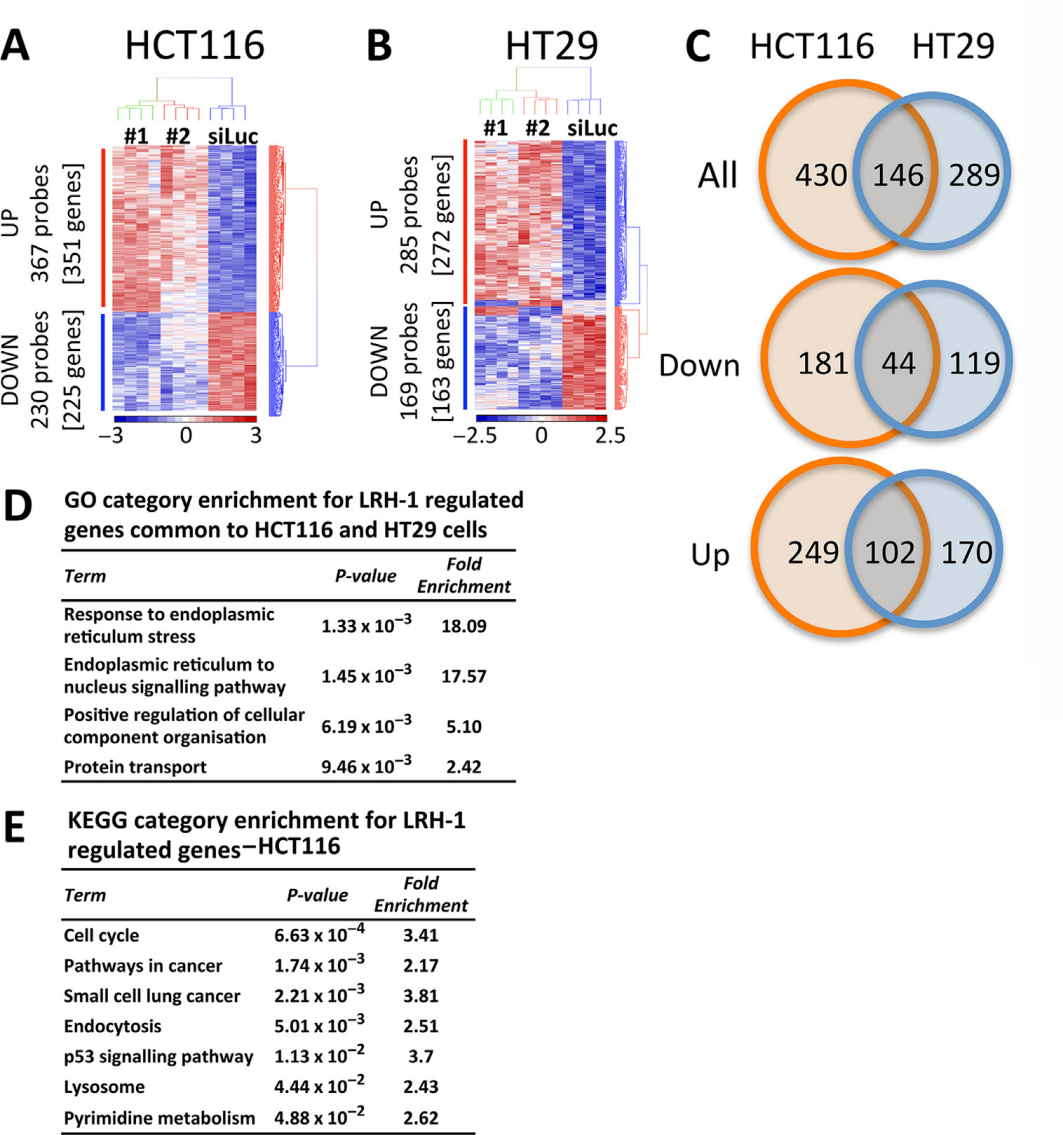
Expression profiling shows that LRH-1 regulates the expression of cell cycle genes in HCT116 cells but not HT29 cells. (**A**, **B**) Gene expression profiling was carried out using four independent replicates of HCT116 cells transfected with siLUC, siLRH-1 #1 or siLRH-1 #2. Shown are differentially expressed genes at *P* < 0.05 corrected for FDR. (**C**) Venn diagram shows the overlap between genes that are differentially expressed upon silencing of LRH-1 in HCT116 (orange) and genes that are differentially expressed upon silencing of LRH-1 in HT29 (blue). (**D**) Gene Ontology (GO) analysis of genes that are LRH-1 regulated in HCT116 and in HT29 cells. (**E**) KEGG Pathway enrichment analysis for LRH-1 regulated genes in HCT116 are shown.

Hierarchical cluster analysis of HCT116 cells identified 597 probes, representing 576 genes, whose expression was significantly altered (*P*-value with false discovery rate (FDR) <0.05) by the LRH-1 siRNAs (Figure 2A). The gene lists are provided in Supplementary Tables S1–S6. Expression of 225 (230 probes) of these genes, including LRH-1 (NR5A2), was significantly reduced, whilst a greater number, 351 genes (367 probes), were up-regulated following LRH-1 knockdown, suggesting that gene repression by LRH-1 is an important feature of its action in HCT116 cells. In HT29 cells, 63% (272 of 435) of LRH-1-regulated genes were up-regulated upon LRH-1 knockdown (Figure 2B), similar to the proportion of the genes (61%) that were up-regulated in HCT116 cells. Real-time RT-PCR analysis for a selection of the identified genes confirmed regulation by LRH-1 in the two cell lines (Supplementary Figure S2).

Comparison of the HCT116 expression profiles with those of HT29 showed that only 25% (146 of 576) of the LRH-1 regulated genes in HCT116 were also regulated in HT29 cells (Figure 2C, Supplementary Tables S1–S6), indicating that the greater part of LRH-1 function is distinct in these cell lines. Pathway enrichment analysis allows the identification of potential signalling pathways and aids in the determination of the putative function of the molecule of interest. To identify signalling pathways that may mediate LRH-1 function in CRC, gene ontology (GO) analyses were undertaken using the online tool DAVID (44,45). Significant GO biological process groups for LRH-1-regulated genes common to both cell lines were related to endoplasmic reticulum processes and protein transport (Figure 2D, Supplementary Table S7). In HT29 cells, LRH-1-regulated genes were especially enriched for processes implicated in protein catabolism, modification, localization and transport (Supplementary Table S7).

In addition to the regulation of protein metabolism, the cell cycle emerged as the most significant GO biological process for LRH-1-regulated genes identified in HCT116 cells. This was confirmed in analysis of Kyoto Encyclopedia of Genes and Genomes (KEGG) pathways in HCT116 cells, which also included the terms ‘pathways in cancer’ and ‘p53 signalling pathway’ (Figure 2E).

### LRH-1 action is associated with regulation of p21 expression in HCT116 cells

**Figure 3.**
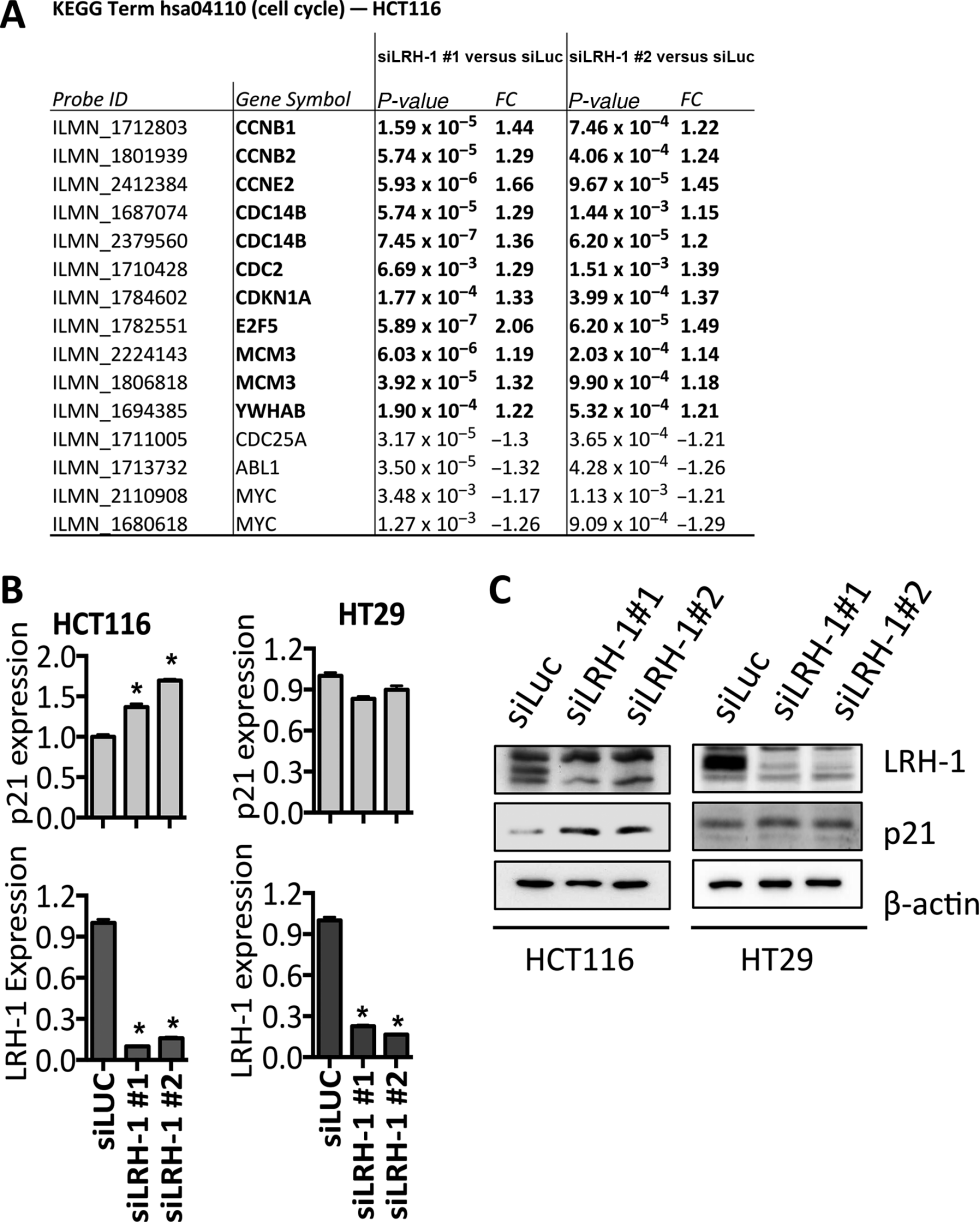
The cyclin dependent kinase (CDK) inhibitor p21 is up-regulated in HCT116 cells following LRH-1 silencing. (**A**) Genes showing LRH-1 regulation in HCT116 that are identified in the KEGG category ‘cell cycle’; FC = fold change. (**B**) Real-time RT-PCR for LRH-1 and p21 (CDKN1A) was performed in HCT116 and HT29 cells following transfection with LRH-1 siRNA. Gene expression was normalised to GAPDH and is shown relative to expression for the control siRNA. The data are the means of six biological replicates and error bars represent the SEM. Statistical significance was calculated by unpaired two-tailed *t*-test, *P < 0.0001. (**C**) Western blotting for LRH-1 and p21 following LRH-1 knockdown in HCT116 and HT29 cells.

Pathway analysis showed enrichment for cell cycle regulation and p53 signalling in HCT116 cells where LRH-1 was silenced. Interestingly, many of the genes associated with the cell cycle, including *CCNB1, CCNB2* and *CCNE2* were up-regulated upon LRH-1 knockdown in HCT116 cells (Figure 3A). Given that LRH-1 knockdown inhibited the growth of HCT116 cells, up-regulation of the p53-regulated cell cycle inhibitor p21, encoded by the *CDKN1A* gene, was especially interesting, since this suggests that promotion of HCT116 cell growth by LRH-1 may involve repression of p21 expression. Real-time RT-PCR and immunoblotting confirmed that p21 is up-regulated following LRH-1 knockdown in HCT116 cells (Figure 3B–C). By contrast, there was no increase in p21 mRNA or protein levels upon LRH-1 knockdown in HT29 cells.

**Figure 4.**
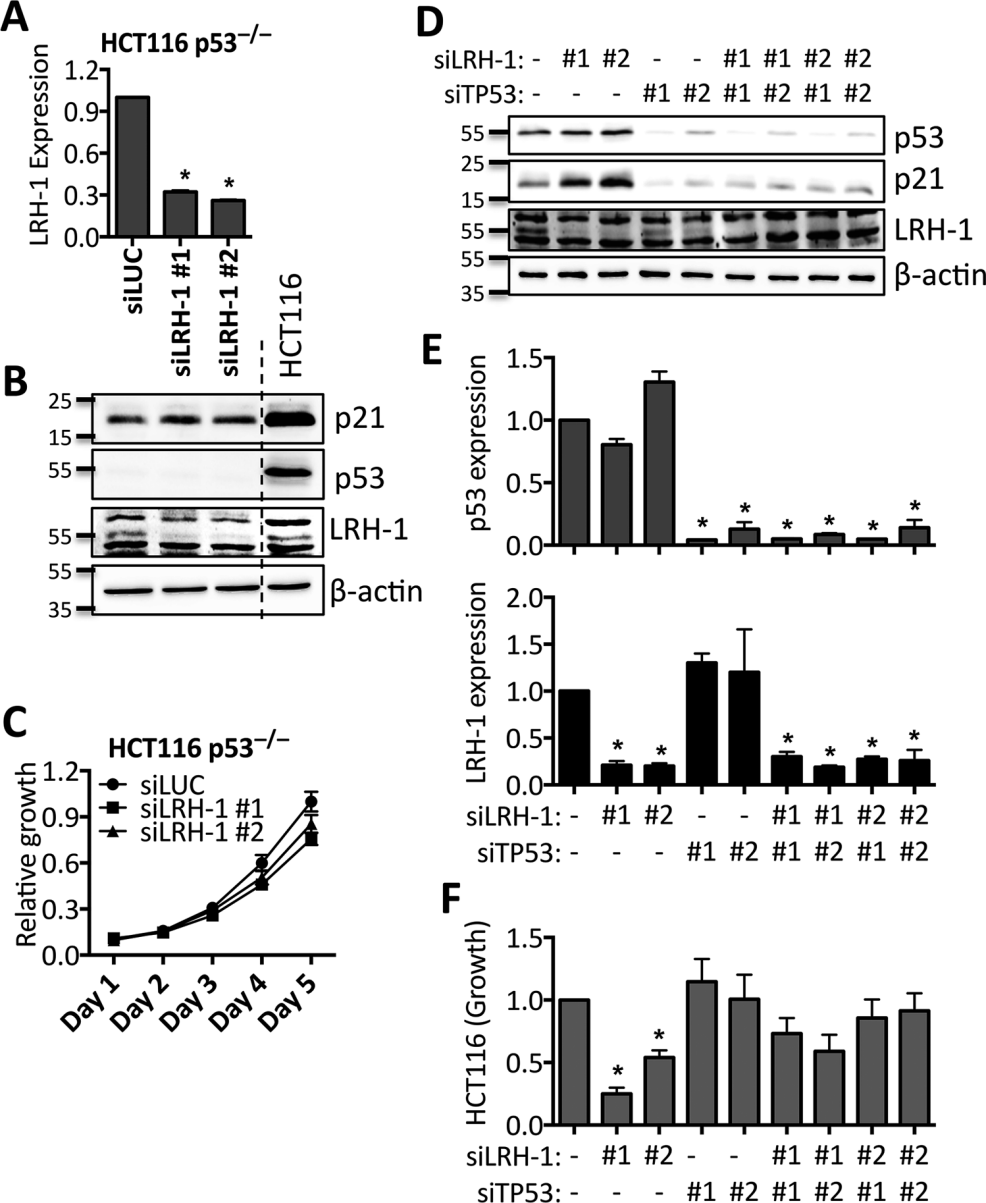
LRH-1 mediated growth inhibition in HCT116 cells requires p53. (**A**, **C**) HCT116 p53^−/−^ cells were transfected with 5 nM siRNA for LRH-1 or siLUC. Real-time RT-PCR (**A**), Western blotting (**B**) and growth (**C**) are shown. (**D**–**F**) HCT116 cells were transfected with 5nM siRNA for LRH-1 and/or 5 nM siRNA for p53 and/or siLUC, to a final siRNA concentration of 10 nM. (**D**) Western blotting following transfection with siRNAs for p53 and/or LRH-1. (**E**) Real-time RT-PCR was performed to confirm knockdown of LRH-1 and p53. (**F**) Growth, as measured by SRB assay, at day five post-transfection, relative to siLUC. Total RNA and protein were prepared 72 h post-transfection. Gene expression data are normalized to GAPDH, and are relative to siLUC. Results shown are the mean of three independent experiments and error bars represent the SEM, * = *P* < 0.01.

While HCT116 cells are wild-type for p53, HT29 cells possess a mutant form of p53 in which arginine-273 is mutated to histidine (R273H) (46). The R273H mutation is classified as a ‘DNA contact’ mutation, which abolishes the ability of p53 to activate transcription of its target genes, including p21 (47,48). It might be hypothesized, therefore, that LRH-1-mediated regulation of p21 occurs via p53, and in HT29 cells, where p53 signalling is abrogated, LRH-1-mediated regulation of p21 is absent. To establish if p53 is indeed required for LRH-1-mediated growth of HCT116 cells, HCT116-p53^−/−^ cells, which lack full-length p53 expression owing to homologous recombination-mediated deletion (38), were transfected with LRH-1 siRNAs. Expression of p21 was not affected and growth was only marginally reduced in the p53-null HCT116 cells (Figure 4A–C).

Knockdown of p53 in HCT116 cells resulted in robust down-regulation of p21 protein expression (Figure 4D), consistent with the fact that p53 is the major transcriptional regulator of p21 (49). While p21 protein expression was increased upon knockdown of LRH-1 only (Figure 4D, E), LRH-1 siRNAs did not increase p21 expression when co-transfected with si-p53. Moreover, although knockdown of p53 did not significantly affect HCT116 cell growth, it was able to rescue the inhibition of HCT116 cell growth by siLRH-1 (Figure 4F).

Similar to our findings for HCT116 and HT29 cells, LRH-1 knockdown inhibited the growth of LOVO cells, which are wild-type for p53 (50), but did not affect growth of HCA46 cells, which are p53 null (Supplementary Figure S3). Moreover, p21 expression was increased by LRH-1 siRNA in LOVO cells, but was unaffected in HCA46 cells, showing that the p53-dependent modulation of p21 expression by LRH-1 is not restricted to HCT116 and HT29 cells.

**Figure 5.**
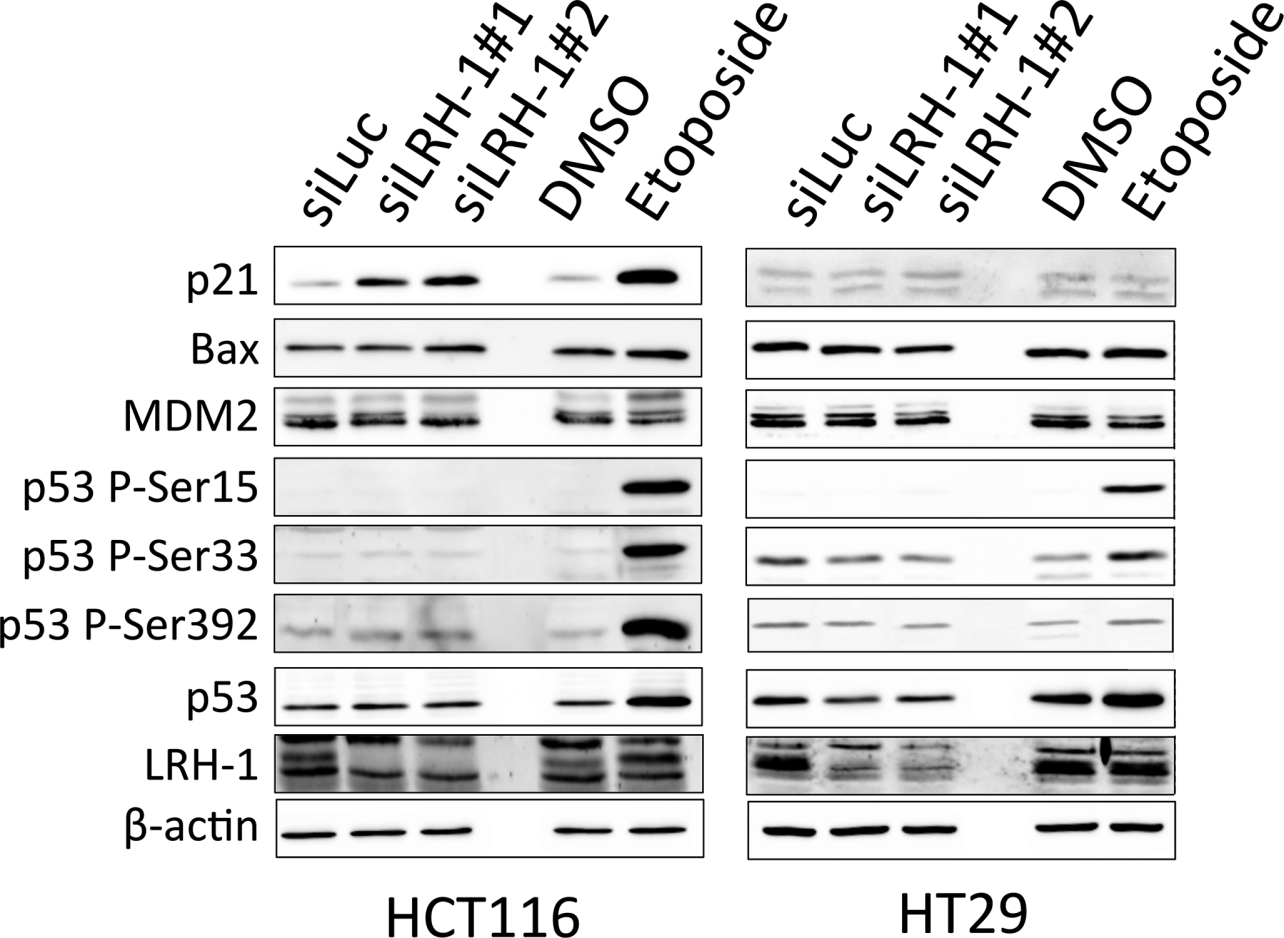
LRH-1 silencing results in up-regulation of p21, but not p53, in HCT116 cells. HCT116 and HT29 cells were transfected with 5nM LRH-1 siRNA or siLUC and protein lysates were prepared 72 h post-transfection. Untransfected HCT116 and HT29 cells were seeded, treated with DMSO or 10 μM etoposide, and protein lysates were prepared after 24 h.

Taken together, the above results show that p21 is an important target for LRH-1 in cells that express non-mutated p53. As p53 is the major transcriptional regulator of p21 (49), we next investigated if LRH-1 regulates the expression of p21 indirectly, via p53. Although gene expression profiling in HCT116 cells did not provide any evidence for the regulation of p53 by LRH-1 at the mRNA level, it is possible that silencing of LRH-1 may result in accumulation of p53 at the protein level. However, no changes in p53 protein levels were observed following transfection of HCT116 and HT29 cells with LRH-1 siRNAs (Figure 5). Stabilization and activation of p53 requires a series of post-translational modifications, and phosphorylation represents a key determinant of p53 activation. Serine-15 and serine-33 of p53 are frequently phosphorylated in response to genotoxic and non-genotoxic stresses, and serine-15 phosphorylation represents a priming event for subsequent modifications (51). No phosphorylation of p53 at serine-15, serine-33, or serine-392 was observed following LRH-1 silencing in HCT116 cells (Figure 5), indicating that p53 is not activated, at least through kinases that phosphorylate these residues. As expected, p53 levels and phosphorylation were stimulated by treatment with the DNA damaging agent etoposide.

Bax, a direct transcriptional target of p53 and a key mediator of p53-dependent apoptosis (52) was increased for siLRH-1 #2, but was unaffected by siLRH-1 #1. Levels of Mdm2, another p53 target gene that is critical for p53 activity, were also unaffected by LRH-1, indicating that inhibition of p53 target gene expression is not a general feature of LRH-1 activity in HCT116 cells.

### Regulation of p21 expression by LRH-1 requires its recruitment to the p21 gene promoter

**Figure 6.**
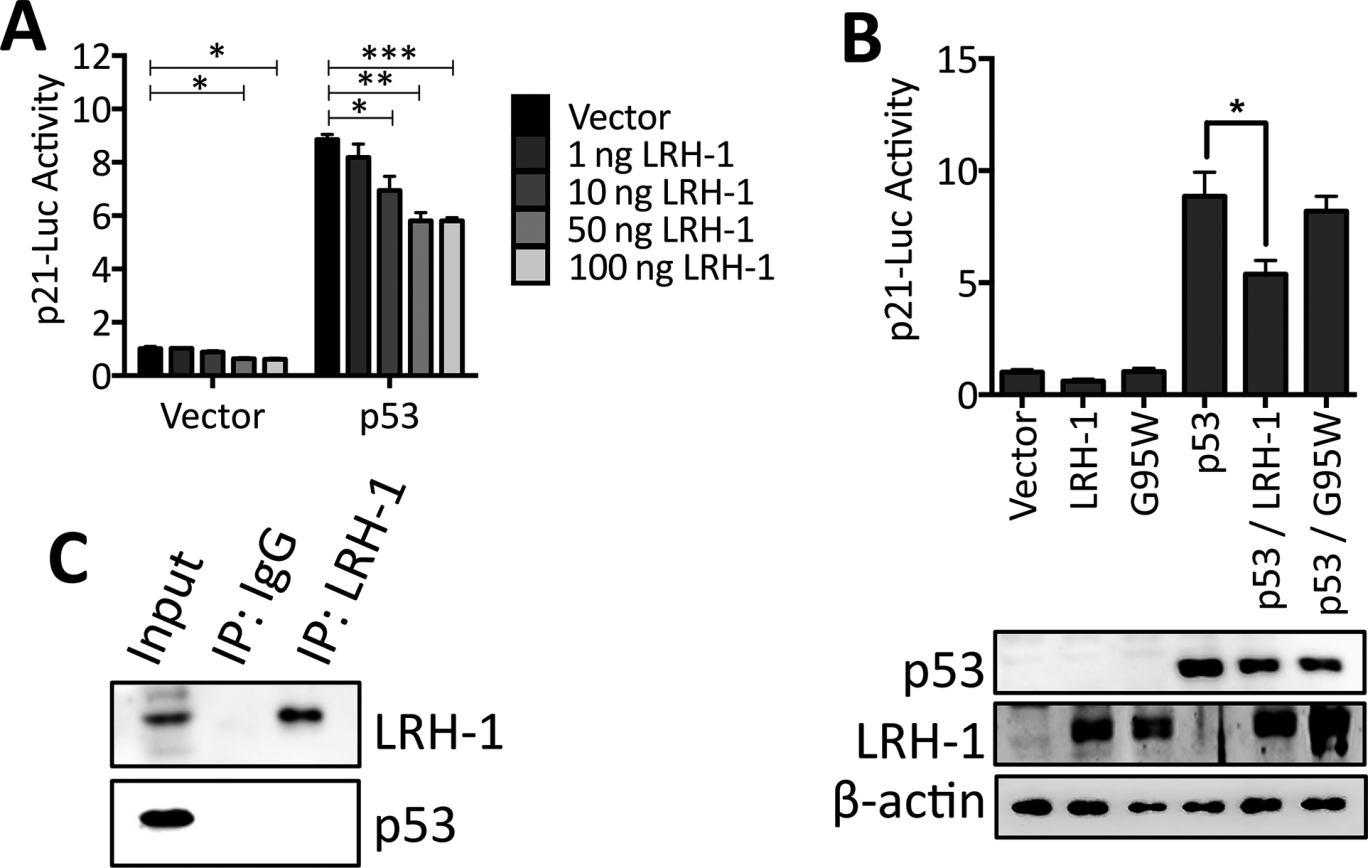
LRH-1 inhibits p53-mediated activation of the p21 promoter. (**A**) The p53-deficient H1299 cells were transfected with p21-Luc, p53, and increasing amounts of LRH-1. p21-Luc activity was corrected for transfection efficiency by normalization to renilla luciferase activity. p21-luc activities are shown relative to the activity for the vector control (n = 3). Statistical significance was calculated by unpaired two-tailed *t*-test: ****P < 0.0001, ***P < 0.001, **P < 0.01, *P < 0.05. (**B**) H1299 cells were transfected with p53, LRH-1 and the G95W LRH-1 mutant. Reporter gene activity was assessed as in part A (n = 3; * *P* < 0.001). Western blotting confirmed expression of LRH-1 and p53. (**C**) HCT116 protein lysates were immunoprecipitated with LRH-1 antibody and immunoblotted for LRH-1 and p53. Input represents 25% of the total volume of lysate used in the immunoprecipitation.

As gene expression profiling and immunoblotting yielded no evidence for LRH-1-mediated regulation of p53 expression or activity, we sought to determine if LRH-1 acts by regulating p53 recruitment to the p21 gene promoter and/or if it directly binds to the p21 promoter. Reporter gene assays with a firefly luciferase gene containing the 2.4 kb promoter region of p21 (39), were performed in p53-null H1299 cells. Transfection of p53 alone potently activated p21-Luc. This p53-mediated activation was inhibited by LRH-1 in a dose-dependent manner (Figure 6A).

Large-scale sequencing projects are being undertaken for the comprehensive cataloguing of genes that are mutated in cancer. Searching the Catalogue of Somatic Mutations in Cancer (COSMIC) database (53) highlights several mutations in the LRH-1 gene in CRC. One of these mutations, a G95W change in the LRH-1 DBD, might be expected to have altered DNA binding. Indeed, this mutation prevented binding of LRH-1 to known LRH-1 binding site sequences from the *TFF1* and *NRIP1* genes (32), as well as the binding site in the *AFP* gene (54) (Supplementary Figure S4). Although wild-type LRH-1 inhibited p53-mediated activation of p21-luc, no inhibition was observed following transfection of the G95W mutant (Figure 6B), indicating that DNA binding is required for LRH-1-mediated repression of p53 transactivation. Interestingly, we found no evidence for direct interaction between LRH-1 and p53 in co-IPs (Figure 6C).

**Figure 7.**
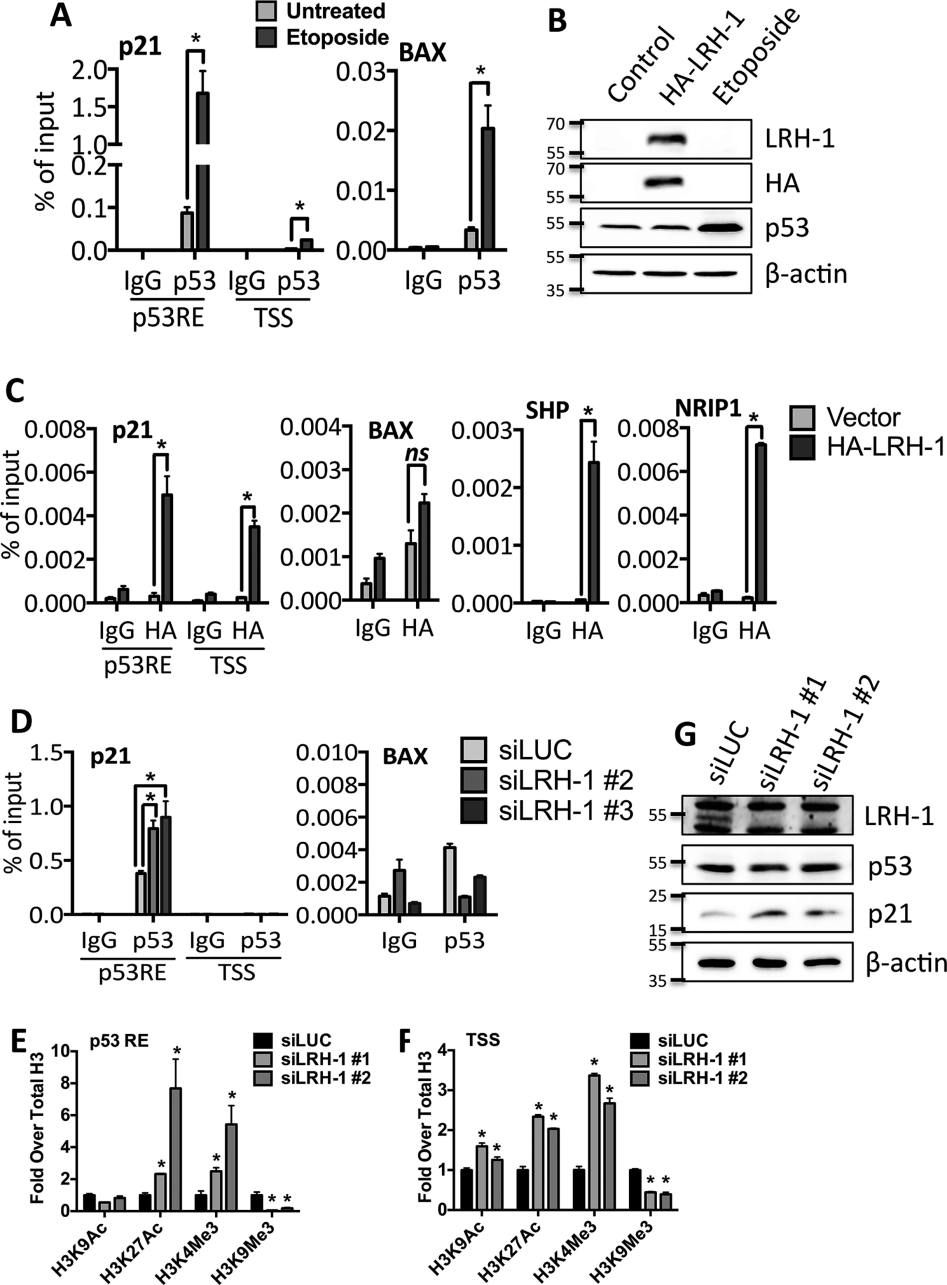
LRH-1 is recruited to the p21 promoter and LRH-1 knockdown enhances p53 recruitment to the p21 promoter. (**A**) HCT116 cells treated with 10 μM etoposide for 24 h were fixed for ChIP for p53. p53 recruitment to the p53RE and TSS regions of the p21 promoter and to the BAX promoter was assessed by qPCR. (**B**) Western blotting for HA-LRH-1 and p53 is also shown. (**C**) HA-LRH-1 transfected HCT116 cells were fixed and harvested after 24 h and ChIP was performed using a HA antibody. HA-LRH-1 recruitment to the p21, BAX, SHP and NRIP1 promoters was assessed by qPCR. (**D**) HCT116 cells transfected with 5 nM siLRH-1 or siLUC were fixed and harvested 24 h post-transfection and p53 ChIP was performed. p53 recruitment to the p21 and BAX gene promoters was determined by qPCR. Bar charts represent the mean of qPCR results obtained from three biological replicates, presented as a percentage of the input DNA and error bars represent SEM. Statistical significance was calculated by unpaired two-tailed *t*-test: *P < 0.01, n.s. = not significant. (**E**, **F**) HCT116 cells were transfected with control siRNA or with two siRNAs for LRH-1. ChIP of histone H3 at acetylation and methylation sites is shown normalized to total H3. *P < 0.05. The data are representative of triplicate experiments, ±SEM. (**G**) Western blotting showing levels of LRH-1, p21 and p53 proteins in the ChIP lysates.

The reporter gene assays suggest that LRH-1 binds to the p21 gene promoter to repress its expression. In ChIP assays, p53 was bound to the p53-response element (p53RE) region of the p21 promoter, and etoposide addition stimulated p53 binding (Figure 7A, B). Similar results were obtained for the BAX promoter. Due to insufficient enrichment of LRH-1 pull-down using commercially available antibodies (32), ChIP for LRH-1 was performed using a HA antibody following transfection of HA-LRH-1 into HCT116 cells. ChIP for HA-LRH-1 demonstrated recruitment of LRH-1 to the p21 promoter at both the p53RE and the transcription start site (TSS) regions (Figure 7C). Enrichment of LRH-1 was also observed at the SHP and NRIP1 promoters, both of which are direct transcriptional targets of LRH-1 (32,55). Immunoblotting confirmed the expression of HA-LRH-1 and the accumulation of p53 following treatment of HCT116 cells with etoposide (Figure 7B).

Knockdown of LRH-1 promoted p53 recruitment to the p21 promoter (Figure 7D), suggesting that LRH-1 prevents p53 recruitment to the p21 gene promoter. LRH-1 knockdown resulted in a marked decrease in levels of the repressive histone H3 mark, H3K9Me3 (Figure 7E, F) and promoted H3 modifications associated with gene activation, specifically acetylation at H3K9, H3K27Ac and methylation of H3K4Me3, indicating that LRH-1 maintains the p21 promoter in a transcriptionally repressive state and that its loss results in chromatin remodelling, which facilitates p21 expression through the action of p53. No significant changes in p53 protein levels were observed (Figure 7G).

## DISCUSSION

An important role for the orphan NR LRH-1 in the development of breast, pancreatic and colon cancer has recently emerged (4,56). In the colon, LRH-1 has been implicated in cell cycle progression and tumourigenesis (35,36). In agreement with work connecting LRH-1 to proliferation in CRC (36), we observed inhibition of HCT116 CRC cell growth following LRH-1 silencing, although the same was not seen for HT29 cells. Global gene expression profiling provides the means of identifying the potential function of LRH-1 in these cells. We used two independent siRNAs for LRH-1 to aid robust identification of LRH-1 regulated genes and performed microarray analysis. This analysis identified 435 and 576 genes that were differentially regulated following LRH-1 siRNA in HT29 and HCT116 cells, respectively. The high numbers of genes identified are consistent with the role of LRH-1 as a transcription factor (4). Only 146 genes were shared between the two cell lines, indicating that LRH-1 regulates the expression of discrete sets of genes in the two lines. It is possible that LRH-1 can regulate different subsets of genes depending on the wider cellular context, i.e. chromosomal instability (CIN), a feature of HT29 cells versus microsatellite instability (MSI), a feature of HCT116 cells (46).

LRH-1 plays key metabolic roles in the liver, including the regulation of cholesterol and bile acid homeostasis (4) and the regulation of glucose metabolism (57). The phosphatidylcholine transfer protein (PCTP) gene, involved in phospholipid exchange (58), was stimulated significantly in HCT116 following LRH-1 knockdown, while SLC10A3, a putative bile acid transporter (59) was down-regulated in HCT116. There was no clear evidence for LRH-1 regulation of additional genes previously identified as LRH-1-regulated in other tissues, which suggests that LRH-1 is not extensively involved in these processes in CRC cells. Interestingly, in line with previous findings (60), there was significant enrichment in both cell lines for biological processes including protein transport, localization and catabolism, thus identifying a novel putative metabolic role for LRH-1 in the intestine and in CRC that deserves detailed investigation.

Previous studies have shown that co-operativity between LRH-1 and β-catenin promotes intestinal cell proliferation, and LRH-1 haploinsufficiency (Lrh^+/−^) in mice is associated with reduced tumourigenesis in the colon (35). Interestingly, the expression of a number of β-catenin target genes associated with cell proliferation, specifically the *CCNB1, CCNB2* and *MYC* genes (61,62), were modulated by LRH-1, suggesting that these genes may be the key mediators of the crosstalk between β-catenin and LRH-1 in CRC. However, *CCND1* and *CCNE1*, previously identified as targets for co-operativity between LRH-1 and β-catenin (25), were not identified as LRH-1 target genes in our microarrays. Bayrer *et al*. (60) showed recently that *CCNE1* expression is reduced upon shRNA mediated LRH-1 knockdown. The authors found that, although *CCNE1* expression was not significantly reduced in HT29 cells, cell growth was modestly reduced, in apparent contrast to our findings. While the differences in our results with those of Bayrer *et al*. are unclear, a role for LRH-1 in CRC cell growth is obvious.

Prominent among the genes identified in our microarray analysis was p21, a key mediator of the p53 response (39). p21 expression is induced by p53 in response to myriad stress stimuli, upon which it binds to and inhibits CDKs, blocking cell cycle progression at G_1_- and S-phases (63,64). p53 regulation of p21 expression occurs via direct DNA binding to two highly conserved p53 response elements in the p21 promoter (39,49). siRNA studies confirmed that LRH-1 regulates the expression of p21 in a p53-dependent manner. Further, we show that LRH-1 inhibits p53 activity on the p21 gene promoter, likely through a mechanism involving the regulation of p53 recruitment to the p53 binding region. Our results indicate that the effects mediated by LRH-1 do not involve modulation of p53 levels, nor do they involve regulation of p53 activity by altered phosphorylation. Silencing of LRH-1 in HT29 cells did not result in up-regulation of p21 or in inhibition of cell growth; these cells possess a mutant, non-transcriptionally active form of p53 (46–48).

**Figure 8.**
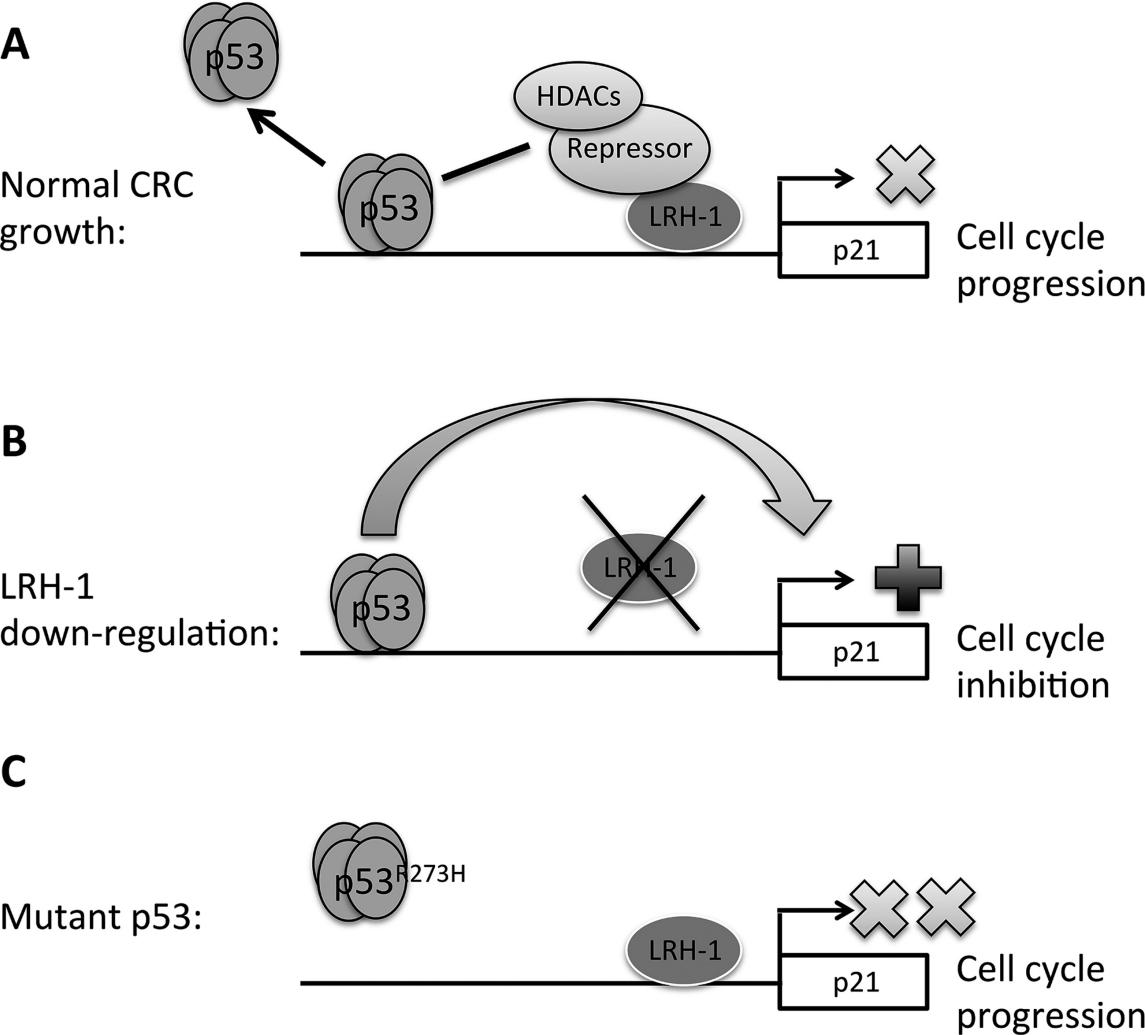
A model for LRH-1-mediated modulation of wild-type p53 signalling at the p21 promoter. (**A**) Recruitment of LRH-1 and associated co-repressors and HDACs to the p21 promoter results in inhibition of p53-mediated transactivation and dissociation of the tetramer from the DNA. This may be facilitated in part through deacetylation of p53 by HDACs, resulting in inhibition of p21 transcription and cell cycle progression. (**B**) LRH-1 down-regulation eliminates its repressive effect on p53, allowing transcription of p21 and cell cycle inhibition. (**C**) In CRC cells harbouring mutant p53, such as the HT29 cell line, p21 transcription is minimal, and recruitment of LRH-1 to the gene promoter has no further effect.

A mechanism to explain the modulation of p53 signalling by LRH-1 involves a form of transcriptional interference, mediated through the concomitant recruitment of one or more co-repressors to the p21 promoter (Figure 8). ChIP and reporter gene studies showed that LRH-1 is recruited to the p21 promoter region. Moreover, a LRH-1 mutant defective for DNA binding did not inhibit p53-mediated stimulation of the p21 reporter gene. Analysis of the p21 promoter sequence included in the p21 reporter gene identified potential LRH-1 binding sites at −2605 to −2597 (5′-TGAAGGTGA-3′) and −1208 to −1201 (5′-GAAGTCCA-3′) bp, the latter overlapping with a site previously linked to regulation of p21 by retinoid receptors (65). LRH-1-directed co-repressor recruitment could exert its influence on p53 by inhibiting p53 transactivation and promoting dissociation of p53 from DNA. The NR co-repressor SHP, which is bound preferentially by LRH-1 (13), interacts with LRH-1 on DNA and abolishes its transactivation ability (66,67). Interestingly, recent evidence suggests that SHP can regulate levels of p53 by augmenting the ubiquitination and degradation of p53 by Mdm2 (68). However, as we have been unable to detect SHP mRNA expression in HCT116 cells (data not shown), it is unlikely that SHP is the co-repressor responsible for this effect. Nor did we observe LRH-1 dependent changes in p53 levels. Other possibilities include the LRH-1 co-repressors SMRT (69), Dax-1 (70) and Prox1 (71), or the NR co-repressor NRIP1, which recruits transcriptional co-repressors of the C-terminal binding protein (CtBP) family, as well as histone deacetylases (HDACs) (72). LRH-1 knockdown resulted in a reduction in levels of the transcription repressive histone H3 modification at lysine-9 and an increase in H3 acetylation, indicating that regulation of p21 by LRH-1 does indeed involve the recruitment of transcriptional co-repressor complexes to the p21 promoter. Additionally, a recent study has identified p21 as a LRH-1 regulated gene in breast cancer cells (73), that involves an additional LRH-1 binding region 62 kb upstream of the p21 gene.

In summary, we demonstrate the importance of LRH-1 in the regulation of CRC cell growth. We provide evidence for modulation of p53 signalling at the p21 promoter, mediated by LRH-1, and propose a model in which LRH-1, in complex with co-repressors, suppresses p53 action at the p21 gene, allowing CRC cells to evade cell cycle arrest mediated by p21. The mutation or loss of p53 is known to be a relatively late event in colorectal tumourigenesis and tends to occur alongside the transition from benign to malignant growth (74). Therefore, it is possible that prior to p53 mutation, LRH-1 allows early adenomas to evade p53-mediated cell cycle arrest induced by pre-malignant changes, such as excessive Wnt pathway activation. The requirement for LRH-1 in the growth of CRC harbouring wild-type p53 makes it a potential drug target, a possibility that undoubtedly warrants further investigation and one that should become amenable to testing with small molecule selective inhibitors of LRH-1 that are actively being sought (75–77).

## Supplementary Material

SUPPLEMENTARY DATA
