# Supplementary material for: LRH-1 drives colon cancer cell growth by repressing the expression of the CDKN1A gene in a p53-dependent manner
Source: Nucleic Acids Res. 2015 Sep 22;44(2):582–94. doi: 10.1093/nar/gkv948 (PMC4737183; doi:10.1093/nar/gkv948)
Supplement: SUPPLEMENTARY DATA [file supp_44_2_582__index.html]

LRH-1 drives colon cancer cell growth by repressing the expression of the CDKN1A gene in a p53-dependent manner — LRH-1 drives colon cancer cell growth by repressing the expression of the CDKN1A gene in a p53-dependent manner — SUPPLEMENTARY DATA 

# LRH-1 drives colon cancer cell growth by repressing the expression of the *CDKN1A* gene in a p53-dependent manner

## SUPPLEMENTARY DATA

- SUPPLEMENTARY DATA
- SUPPLEMENTARY DATA
- SUPPLEMENTARY DATA
- SUPPLEMENTARY DATA
- SUPPLEMENTARY DATA
- SUPPLEMENTARY DATA
- SUPPLEMENTARY DATA
- SUPPLEMENTARY DATA
- SUPPLEMENTARY DATA
- SUPPLEMENTARY DATA
- SUPPLEMENTARY DATA
