# Supplementary figures and images for: LRH-1 drives colon cancer cell growth by repressing the expression of the CDKN1A gene in a p53-dependent manner
Source: Nucleic Acids Res. 2015 Sep 22;44(2):582–94. doi: 10.1093/nar/gkv948 (PMC4737183; doi:10.1093/nar/gkv948)

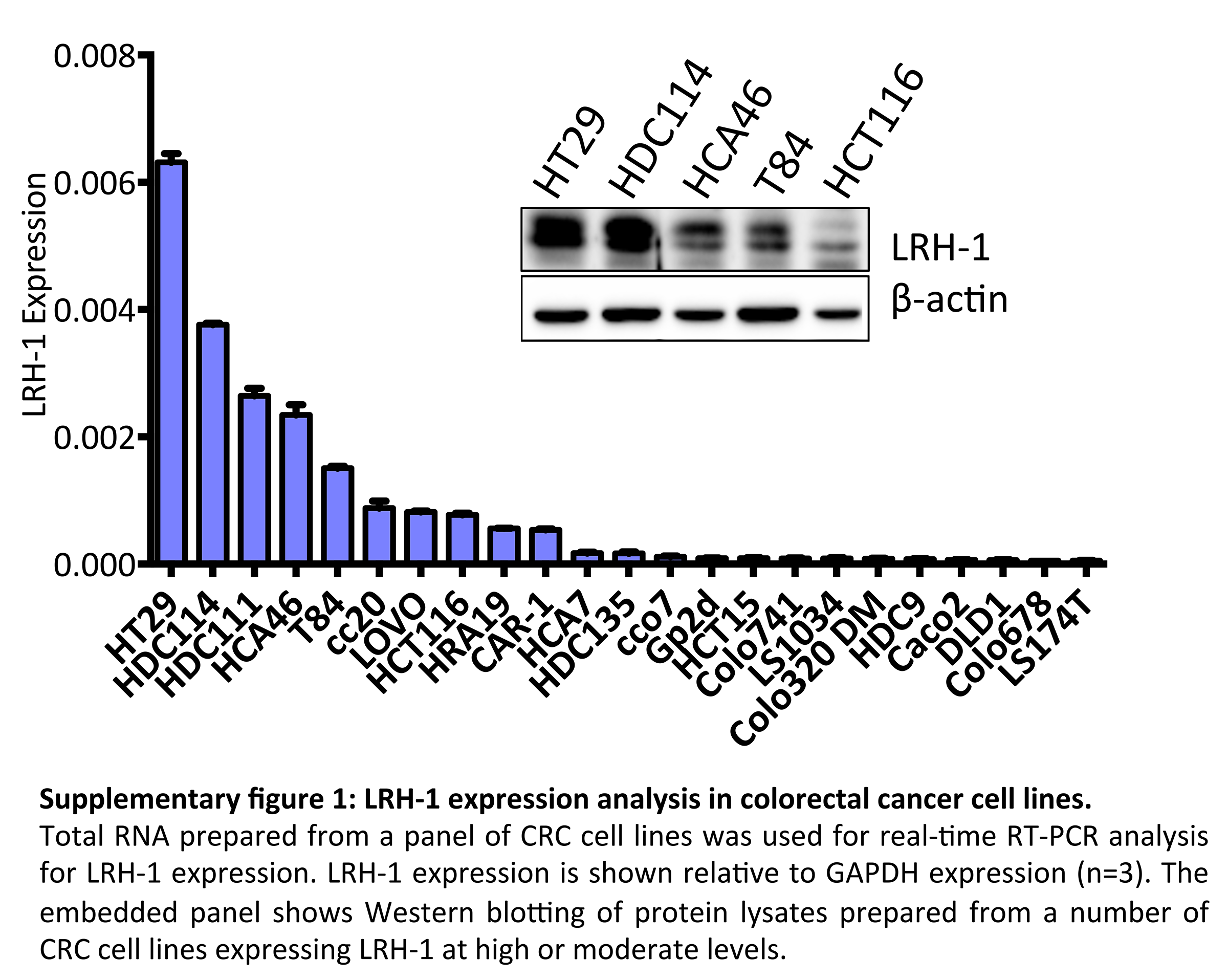

Supplement: SUPPLEMENTARY DATA [file supp_gkv948_nar-01236-x-2015-File010.tif]

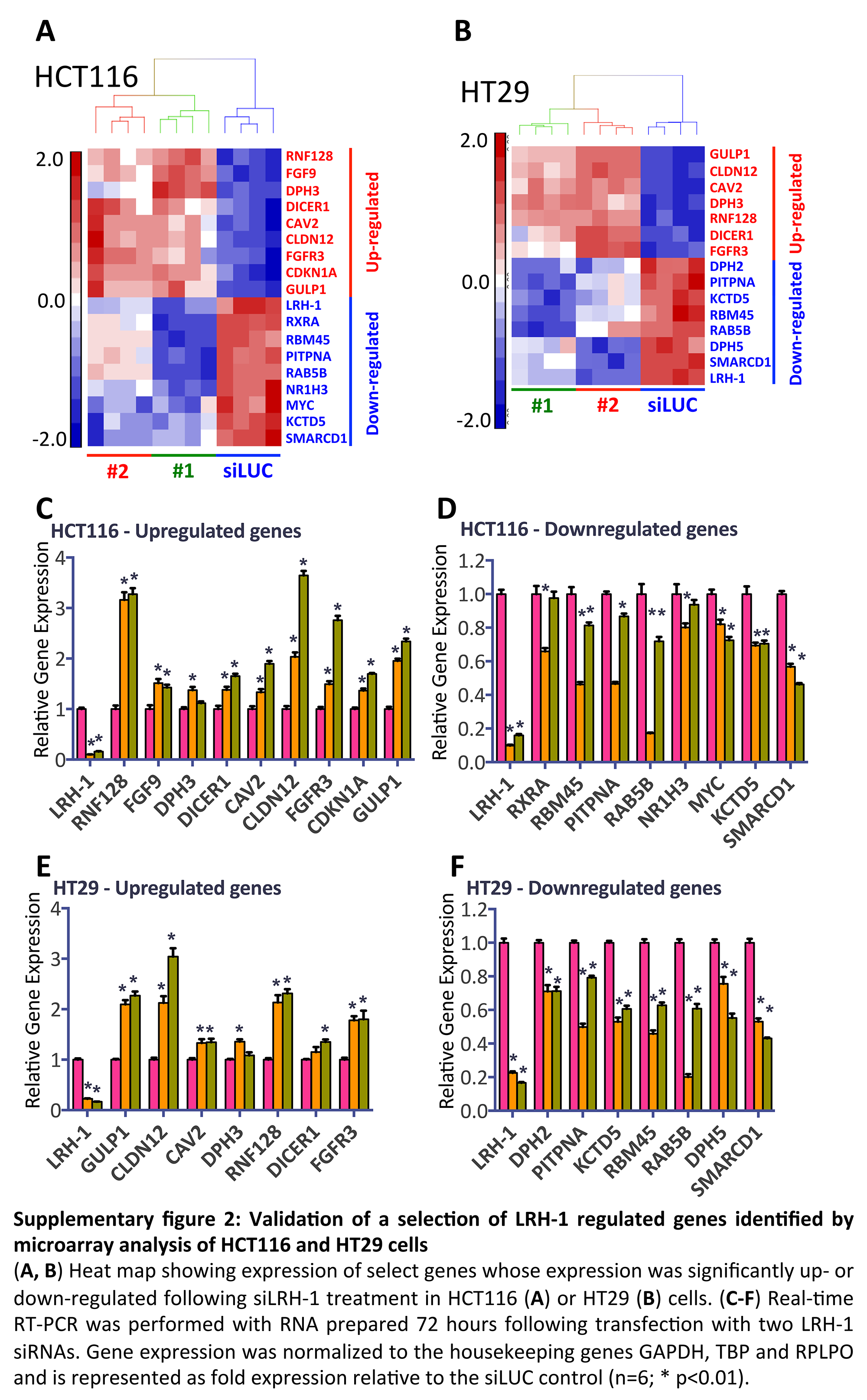

Supplement: SUPPLEMENTARY DATA [file supp_gkv948_nar-01236-x-2015-File011.tif]

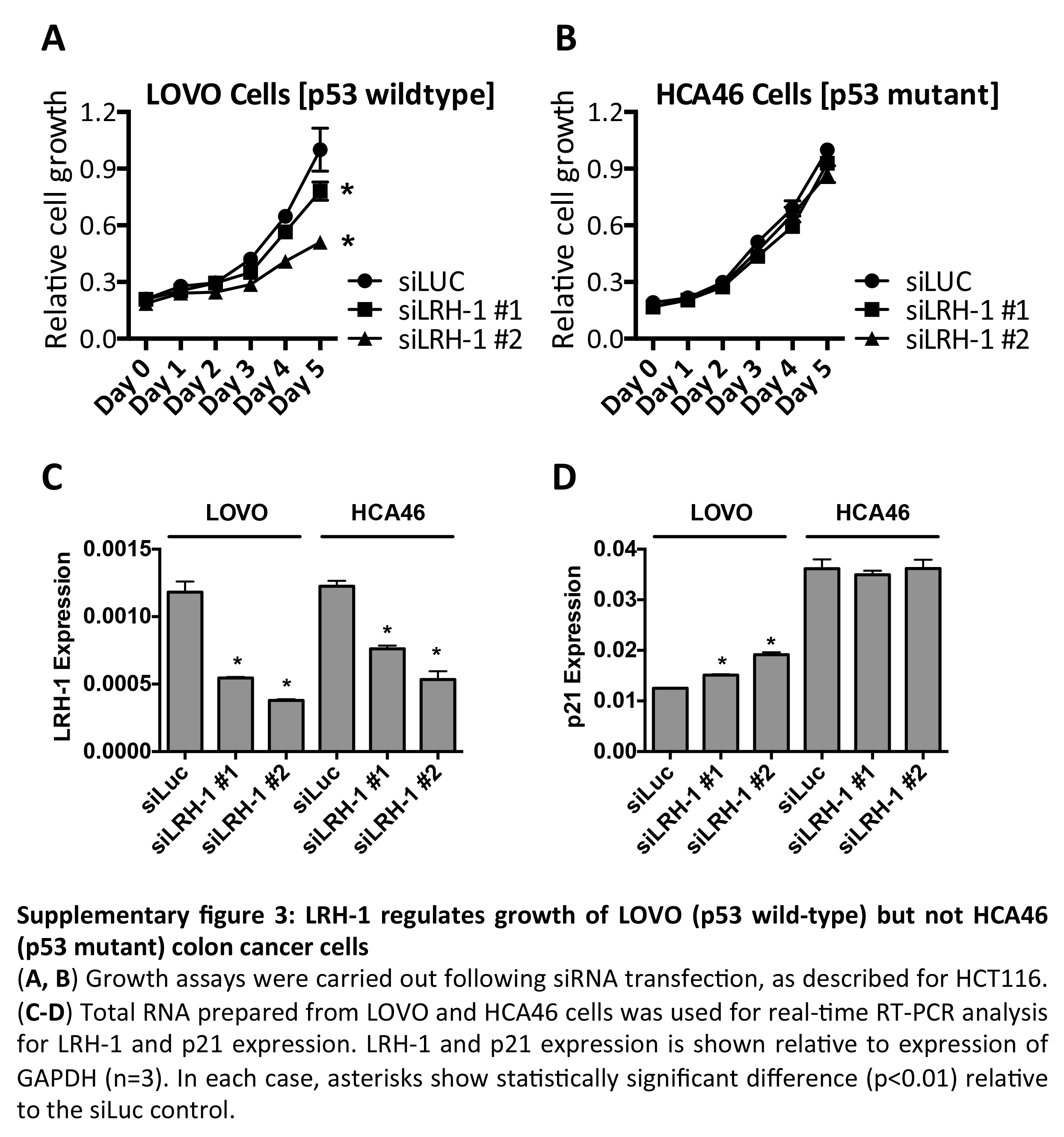

Supplement: SUPPLEMENTARY DATA [file supp_gkv948_nar-01236-x-2015-File012.tif]

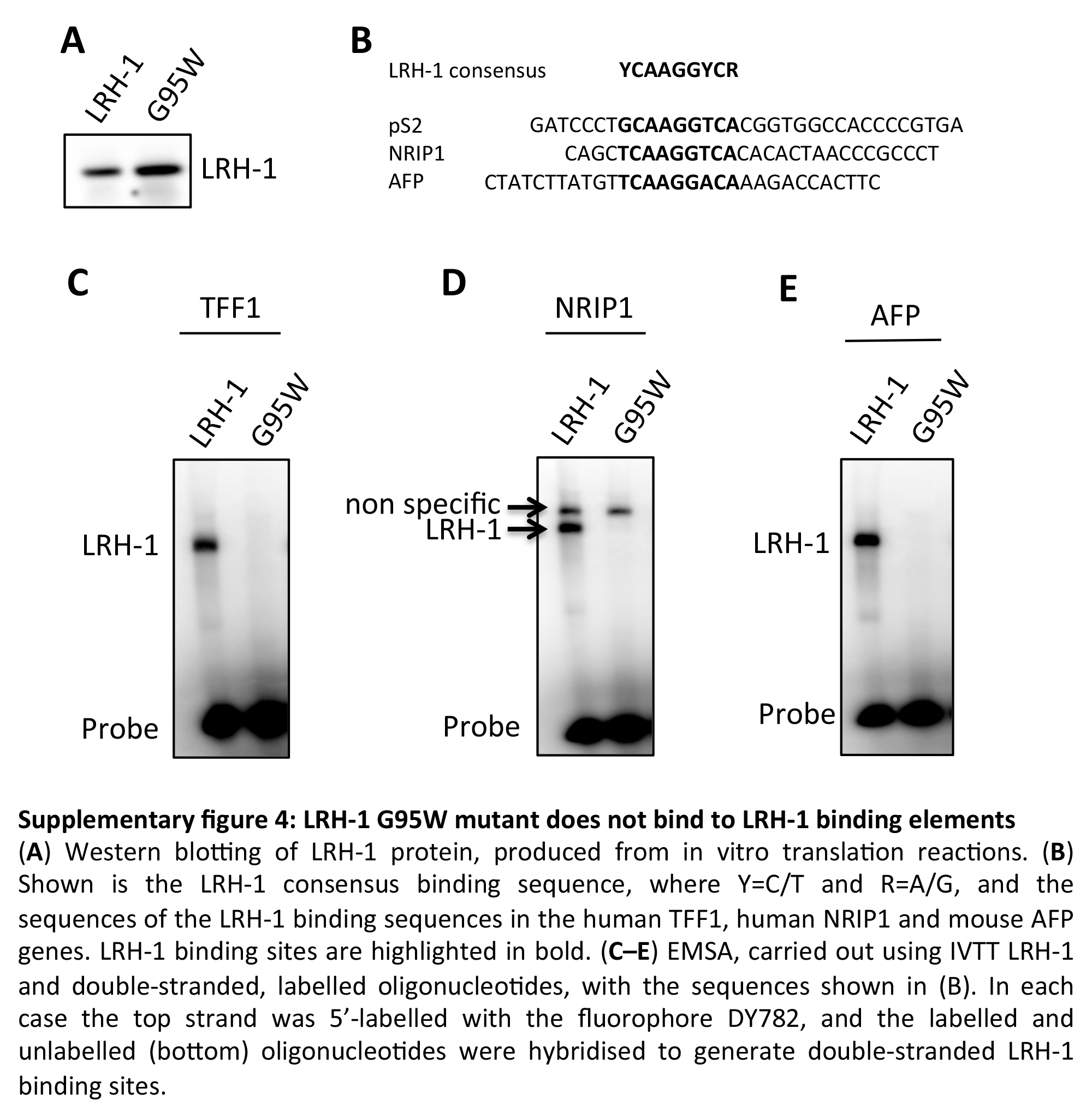

Supplement: SUPPLEMENTARY DATA [file supp_gkv948_nar-01236-x-2015-File013.tif]
