# Supplementary material for: LRH-1 drives colon cancer cell growth by repressing the expression of the CDKN1A gene in a p53-dependent manner
Source: Nucleic Acids Res. 2015 Sep 22;44(2):582–94. doi: 10.1093/nar/gkv948 (PMC4737183; doi:10.1093/nar/gkv948)
Supplement: SUPPLEMENTARY DATA [file supp_gkv948_nar-01236-x-2015-File014.pdf]

Supplementary Table 1. Genes up-regulated in HCT116 AND HT29 following LRH-1 siRNA

		HCT116 siLRH-1 #1 vs siLuc		HCT116 siLRH-1 #2 vs siLuc		HT29 siLRH-1 #1 vs siLuc		HT29 siLRH-1 #2 vs siLuc	
Probeset ID	*Gene Symbol*	*p-value**	*Fold Change*	*p-value**	*Fold Change*	*p-value**	*Fold Change*	*p-value**	*Fold Change*
ILMN_3208881	LOC647597	3.18E-07	1.61	6.20E-05	1.26	1.27E-05	1.50	1.94E-05	1.42
ILMN_3238676	ULBP2	3.18E-07	2.22	1.70E-05	1.66	0.00032209	1.41	0.00305862	1.26
ILMN_1782551	E2F5	5.89E-07	2.06	6.20E-05	1.49	1.64E-05	1.92	0.00132331	1.34
ILMN_2379560	CDC14B	7.45E-07	1.36	6.20E-05	1.20	0.0002023	1.26	0.00254226	1.16
ILMN_2269256	DNAJC12	1.00E-06	1.65	6.30E-05	1.35	0.00078776	1.36	0.00315733	1.27
ILMN_1679912	DPH3	1.00E-06	1.56	0.000207531	1.20	2.33E-06	1.50	5.38E-06	1.38
ILMN_2393046	NHLRC3	2.58E-06	1.68	6.20E-05	1.45	2.68E-06	2.30	1.94E-05	1.74
ILMN_1688322	ADIPOR1	3.80E-06	1.44	0.000195414	1.21	0.00027634	1.35	0.00315733	1.22
ILMN_2349610	DPH3	3.89E-06	1.54	0.000306332	1.23	2.70E-06	1.65	4.47E-06	1.56
ILMN_1803073	DNAJC12	4.47E-06	1.62	0.000288991	1.27	0.00025223	1.30	0.00149433	1.22
ILMN_1781791	PRRG1	4.47E-06	1.81	6.30E-05	1.55	2.26E-05	1.94	0.00143793	1.37
ILMN_2412384	CCNE2	5.93E-06	1.66	9.67E-05	1.45	6.56E-05	1.43	1.94E-05	1.55
ILMN_2106902	CHES1	5.93E-06	1.48	6.30E-05	1.37	0.00079584	1.32	0.00048743	1.35
ILMN_1792712	LOC201725	5.93E-06	1.84	8.83E-05	1.57	0.00162586	1.46	0.00300822	1.40
ILMN_1692260	MAFG	5.93E-06	1.62	0.000573929	1.25	3.63E-05	1.36	0.00033971	1.23
ILMN_1690049	NGDN	5.93E-06	1.47	0.000129007	1.28	0.0003495	1.19	9.17E-05	1.25
ILMN_2075189	SLC35F2	5.95E-06	1.64	6.30E-05	1.51	0.00012318	1.31	0.00010883	1.31
ILMN_1778064	FICD	6.33E-06	1.66	9.67E-05	1.44	2.63E-05	1.49	3.35E-05	1.46
ILMN_3249006	LOC100133888	6.38E-06	1.53	0.000378516	1.25	0.00013401	1.33	0.00319074	1.18
ILMN_1804822	SRXN1	8.17E-06	1.68	0.000207531	1.37	4.31E-05	1.40	0.00149433	1.20
ILMN_1656111	MYLIP	8.50E-06	1.50	0.000128227	1.34	0.00308655	1.14	0.00253656	1.15
ILMN_1851492	HS.374278	8.83E-06	1.79	9.67E-05	1.56	3.94E-06	1.77	1.09E-05	1.60
ILMN_1775742	RNF128	9.49E-06	1.79	9.96E-05	1.56	4.82E-06	1.71	3.99E-06	1.75
ILMN_1790577	SLC35F2	1.11E-05	1.76	6.30E-05	1.67	2.26E-05	1.37	1.94E-05	1.36
ILMN_2181125	NAPB	1.15E-05	1.78	9.67E-05	1.60	0.00010424	1.35	4.30E-05	1.41
ILMN_1698258	DNAJC8	1.20E-05	1.40	6.62E-05	1.35	7.58E-05	1.26	1.47E-05	1.36
ILMN_3253456	FNDC3B	1.20E-05	1.47	9.67E-05	1.37	4.67E-05	1.36	1.94E-05	1.43
ILMN_1676625	SS18L1	1.20E-05	1.33	0.000195414	1.21	0.00046902	1.27	0.0005737	1.26
ILMN_2313926	CDC42SE2	1.30E-05	1.88	0.00014186	1.62	1.64E-05	1.62	4.21E-05	1.48
ILMN_1793651	UBE2N	1.30E-05	1.41	0.000546422	1.21	0.00010424	1.26	0.00015267	1.24
ILMN_1772455	HDAC3	1.48E-05	1.56	0.000195414	1.37	2.48E-05	1.43	2.41E-05	1.41
ILMN_1729288	C1QTNF6	1.62E-05	1.38	0.000204053	1.26	0.00101546	1.20	0.00137112	1.19
ILMN_1723467	ITGB1	1.62E-05	1.52	0.000134769	1.40	0.00023226	1.23	0.00310325	1.14
ILMN_1677829	SLC9A6	1.62E-05	1.49	9.67E-05	1.42	8.34E-05	1.35	6.92E-05	1.36
ILMN_1796925	CXADR	1.65E-05	1.39	0.000176204	1.29	6.60E-05	1.25	0.00024823	1.20
ILMN_1759023	WFS1	1.65E-05	1.50	0.000573929	1.26	0.00037137	1.38	0.00060298	1.35
ILMN_1795963	OKL38	1.89E-05	1.43	0.000195414	1.31	4.31E-05	1.33	0.0007572	1.19
ILMN_1728202	TMEM22	2.21E-05	1.17	0.000207531	1.13	0.00018393	1.27	0.00119961	1.19
ILMN_1689327	LOC730534	2.42E-05	1.29	0.000251061	1.20	0.00227629	1.10	0.00038755	1.14
ILMN_1689817	LCOR	2.42E-05	1.57	0.000195414	1.41	0.00186343	1.28	0.00044965	1.38
ILMN_1678087	MAP3K4	2.72E-05	1.43	0.000195414	1.33	0.00130408	1.17	0.00127345	1.18
ILMN_1730118	ZNF644	2.74E-05	1.70	0.00015985	1.57	0.00178777	1.26	0.00018408	1.41
ILMN_1797310	ATP6V1D	3.45E-05	1.43	0.000731793	1.24	0.00023226	1.24	9.04E-05	1.29
ILMN_1744963	ERO1L	4.14E-05	1.66	0.000195414	1.53	1.64E-05	1.65	6.84E-06	1.75
ILMN_1660551	CRAMP1L	4.16E-05	1.15	0.000691137	1.09	0.00142356	1.13	0.00169478	1.12
ILMN_1660063	POLE4	4.44E-05	1.31	0.00043241	1.21	0.00186343	1.17	0.00305862	1.16
ILMN_1685602	TMEM41A	4.50E-05	1.58	0.000188594	1.50	4.24E-05	1.44	5.21E-05	1.41
ILMN_2313901	PAM	5.38E-05	1.28	0.000221667	1.23	0.00069439	1.33	0.00317162	1.24
ILMN_3249240	C4orf46	5.66E-05	1.79	0.000221667	1.64	4.38E-05	1.31	1.94E-05	1.37
ILMN_1788062	SH3GL1	6.05E-05	1.32	0.00014186	1.32	2.47E-05	1.24	5.11E-05	1.20
ILMN_1664449	ALG5	7.03E-05	1.37	0.000573929	1.25	0.00018819	1.19	3.35E-05	1.26
ILMN_2062112	ZC3H15	7.93E-05	1.39	0.000743398	1.25	0.00010424	1.24	3.42E-05	1.30
ILMN_1839719	HS.14555	0.00010717	1.42	0.000643596	1.30	0.00041486	1.21	0.00152039	1.17
ILMN_1738712	GPR180	0.00013275	1.31	0.000306019	1.27	0.00046728	1.27	0.00011658	1.35
ILMN_1724376	C2orf30	0.00014385	1.43	0.00135093	1.27	2.47E-05	1.43	9.58E-05	1.31
ILMN_1679071	MTX3	0.00015638	1.50	0.000365827	1.43	0.0002023	1.22	4.30E-05	1.30
ILMN_1763523	HARS	0.00017557	1.45	0.000546422	1.36	2.93E-06	1.53	3.10E-06	1.54
ILMN_1784206	LOC653308	0.00019577	1.73	0.000151134	1.88	4.31E-05	1.29	3.99E-06	1.47
ILMN_1664920	C19orf12	0.00019924	1.19	0.000691137	1.15	0.00020501	1.24	0.00114725	1.18

ILMN_2218935	GPR37	0.00020269	1.24	0.00128634	1.17	0.00021005	1.30	0.00116767	1.22
ILMN_1765204	ST13	0.00021153	1.36	0.000573929	1.30	0.00074026	1.18	0.00123527	1.17
ILMN_1763634	PEX14	0.00021364	1.27	0.00107602	1.20	0.00320075	1.19	0.00149433	1.23
ILMN_3178302	FNDC3B	0.00023395	1.54	0.00043241	1.49	7.52E-05	1.38	0.00023105	1.31
ILMN_1686750	MGEA5	0.00023395	1.25	0.000771131	1.20	0.00246872	1.15	0.00018777	1.25
ILMN_1784333	SECISBP2L	0.00025849	1.16	0.00072	1.13	0.00275901	1.19	0.00064726	1.26
ILMN_1815261	PDIA4	0.00031693	1.28	0.00121198	1.21	0.00034252	1.15	0.00137112	1.12
ILMN_1802690	GULP1	0.00032271	1.53	0.000236222	1.60	1.23E-06	1.58	6.74E-08	1.80
ILMN_1797728	HMGCS1	0.00032271	1.26	0.000365827	1.27	0.00082734	1.30	0.00029539	1.37
ILMN_1725642	SUMO3	0.00044721	1.65	0.000195414	1.84	4.87E-05	1.37	7.54E-05	1.34
ILMN_1687533	SEMA4D	0.00046152	1.23	0.000611453	1.21	1.27E-05	1.31	0.00026634	1.16
ILMN_2402798	AP2M1	0.00047336	1.14	0.000422921	1.15	8.34E-05	1.18	0.00029539	1.15
ILMN_1787657	CLDN12	0.0004792	1.92	0.000176204	2.34	1.59E-06	2.21	8.93E-08	2.86
ILMN_1748968	ATG10	0.00048086	1.28	0.000221667	1.34	7.88E-05	1.49	0.00016048	1.42
ILMN_1661940	CAMTA1	0.00048236	1.34	0.000689614	1.32	0.00021005	1.27	0.00025356	1.26
ILMN_1671048	ZNF644	0.00048236	1.35	0.000912102	1.30	0.00123178	1.24	0.00048743	1.29
ILMN_1658835	CAV2	0.00056092	1.50	0.000195414	1.69	2.93E-06	1.84	1.76E-06	2.02
ILMN_1735680	TMEM30A	0.00056338	1.24	0.000573061	1.24	0.00024996	1.35	0.00109686	1.27
ILMN_1653134	TMEM188	0.00064754	1.40	0.000576904	1.40	0.00154738	1.24	0.00010179	1.41
ILMN_2412294	GNB5	0.00070519	1.34	0.000573929	1.35	0.00021971	1.36	5.11E-05	1.48
ILMN_2150402	TMEM64	0.00081075	1.26	0.000448929	1.29	0.00088281	1.29	9.58E-05	1.45
ILMN_1807662	IGF2R	0.00098431	1.27	0.000834387	1.27	0.00111808	1.16	0.00029539	1.21
ILMN_1702821	TTLL7	0.00098431	1.17	0.00151808	1.15	0.00111552	1.18	0.00036452	1.22
ILMN_1669905	DCP2	0.00125877	1.59	0.000207531	1.93	2.49E-05	1.42	2.61E-06	1.71
ILMN_1690844	LOC387820	0.00136821	1.14	0.000984295	1.15	0.00011986	1.22	4.30E-05	1.27
ILMN_1803941	TBC1D15	0.00144429	1.24	0.00135093	1.24	4.38E-05	1.18	1.36E-05	1.23
ILMN_1898682	HS.556018	0.00154387	1.31	0.0012044	1.32	0.00111808	1.30	0.00061186	1.34
ILMN_1726720	NUSAP1	0.00159041	1.23	0.00043241	1.30	0.00281738	1.13	5.51E-06	1.44
ILMN_2290118	MEGF9	0.00160762	1.21	0.000546422	1.26	5.08E-05	1.25	5.51E-06	1.39
ILMN_2052891	PKD2	0.00179588	1.29	0.000526815	1.38	0.00192235	1.18	0.00010179	1.31
ILMN_2360730	CAV2	0.00180792	1.43	0.000387847	1.61	5.16E-05	1.50	2.06E-05	1.61
ILMN_1651557	KDELC2	0.00221062	1.30	0.000345266	1.45	1.27E-05	1.43	1.09E-05	1.42
ILMN_1710738	RC3H2	0.00221062	1.21	0.000860356	1.25	0.00037137	1.18	9.46E-05	1.24
ILMN_3306730	RBM47	0.00241768	1.29	0.00105907	1.33	4.31E-05	1.39	3.42E-05	1.40
ILMN_2065783	EXOC2	0.00283124	1.15	0.0003526	1.22	3.02E-05	1.21	5.38E-06	1.29
ILMN_1700915	BMI1	0.00324046	1.21	0.00142522	1.24	0.00016386	1.24	6.48E-05	1.28
ILMN_1723123	FGFR3	0.00328021	1.49	0.000221667	1.94	2.33E-06	1.64	4.21E-08	2.48
ILMN_3224926	RBM47	0.00392855	1.21	0.000402994	1.33	2.59E-05	1.36	3.49E-05	1.33
ILMN_1664608	INPP5A	0.00406363	1.26	0.00043241	1.41	0.00012734	1.34	1.94E-05	1.50
ILMN_1758941	REEP5	0.00406363	1.14	0.000490912	1.22	0.00054967	1.19	9.17E-05	1.27
ILMN_1680130	DYM	0.00471312	1.13	0.000526815	1.20	0.00046349	1.19	0.00051233	1.19
ILMN_1752927	KIAA1600	0.00471312	1.43	0.000387847	1.75	0.00080518	1.15	2.61E-06	1.47
ILMN_1714083	KLHL8	0.00602336	1.18	0.00151808	1.24	0.00080518	1.21	0.00046443	1.24
ILMN_1757732	OSGIN2	0.00700076	1.20	0.0012044	1.28	1.27E-05	1.18	5.38E-06	1.21

* False discovery rate (FDR) adjusted p-value
