# Supplementary material for: LRH-1 drives colon cancer cell growth by repressing the expression of the CDKN1A gene in a p53-dependent manner
Source: Nucleic Acids Res. 2015 Sep 22;44(2):582–94. doi: 10.1093/nar/gkv948 (PMC4737183; doi:10.1093/nar/gkv948)
Supplement: SUPPLEMENTARY DATA [file supp_gkv948_nar-01236-x-2015-File015.pdf]

Supplementary Table 2. Genes that are down-regulated in HCT116 AND HT29 following LRH-1 siRNA

		**HCT116 siLRH-1 #1 vs siLuc**		**HCT116 siLRH-1 #2 vs siLuc**		**HT29 siLRH-1 #1 vs siLuc**		**HT29 siLRH-1 #2 vs siLuc**	
Probeset ID	*Gene Symbol*	*p-value**	*Fold Change*	*p-value**	*Fold Change*	*p-value**	*Fold Change*	*p-value**	*Fold Change*
ILMN_1752582	RAB5B	3.37E-07	-3.36	0.000333337	-1.49	7.90E-06	-2.81	0.00329919	-1.39
ILMN_1680378	RBM45	6.04E-07	-2.10	0.000333337	-1.32	7.90E-06	-1.58	0.00010919	-1.32
ILMN_1789349	UBQLN4	1.18E-06	-1.72	0.000243463	-1.38	1.55E-05	-1.40	3.02E-05	-1.35
ILMN_1698307	DBNL	1.53E-06	-1.51	0.000298317	-1.25	0.00011419	-1.19	0.00193344	-1.11
ILMN_1738989	GOLSYN	1.53E-06	-1.86	0.000358049	-1.33	0.00019037	-1.34	0.00329919	-1.20
ILMN_1715804	PITPNA	4.44E-06	-1.62	0.000943181	-1.21	1.49E-05	-1.44	0.00031127	-1.23
ILMN_1796235	CIRH1A	7.40E-06	-1.39	0.00144366	-1.14	7.43E-05	-1.19	0.00010919	-1.18
ILMN_1680781	C14orf135	1.28E-05	-1.57	0.000462786	-1.29	8.98E-06	-1.47	0.00077146	-1.19
ILMN_1682694	LOC203547	1.37E-05	-1.59	0.00144366	-1.23	0.00011419	-1.51	0.00012203	-1.52
ILMN_1662130	LOC730316	1.37E-05	-1.46	0.000243463	-1.39	0.00040715	-1.23	0.00012938	-1.29
ILMN_1753008	REXO1	1.37E-05	-1.55	0.000333337	-1.33	2.88E-05	-1.40	0.00188335	-1.18
ILMN_1672504	PDXK	1.55E-05	-1.44	0.000298317	-1.30	2.32E-05	-1.30	5.46E-06	-1.38
ILMN_1666933	ASH2L	1.74E-05	-1.21	0.000511344	-1.12	4.25E-05	-1.22	0.00037391	-1.15
ILMN_1684306	S100A4	1.74E-05	-1.23	0.00053437	-1.13	0.00043556	-1.12	6.92E-05	-1.18
ILMN_2044085	RQCD1	1.87E-05	-1.36	0.000432421	-1.22	8.29E-05	-1.34	0.00077146	-1.23
ILMN_1801616	EMP1	1.93E-05	-1.39	0.00036522	-1.25	5.88E-05	-1.26	5.46E-06	-1.40
ILMN_1697218	MED22	2.19E-05	-1.39	0.000298317	-1.31	0.00019037	-1.34	0.00031127	-1.32
ILMN_1811303	NR5A2	3.09E-05	-1.34	0.000426981	-1.23	9.68E-07	-2.52	7.10E-08	-3.47
ILMN_1711005	CDC25A	3.17E-05	-1.30	0.00036522	-1.21	1.74E-05	-1.27	1.04E-05	-1.29
ILMN_3276656	LOC728249	4.42E-05	-1.31	0.00053437	-1.21	0.00274765	-1.22	0.00240613	-1.22
ILMN_1808821	COMMD9	4.72E-05	-1.33	0.000298317	-1.30	6.82E-05	-1.42	0.00037391	-1.30
ILMN_1658830	C10orf26	4.96E-05	-1.52	0.00036522	-1.40	1.03E-05	-1.39	5.46E-06	-1.40
ILMN_2366654	NR5A2	5.12E-05	-1.31	0.000450212	-1.23	3.74E-06	-2.36	1.49E-07	-3.47
ILMN_1693108	RUVBL1	5.17E-05	-1.24	0.000604671	-1.17	0.00136604	-1.13	0.00020649	-1.19
ILMN_1802456	DCTD	5.40E-05	-1.44	0.000367253	-1.34	0.00045672	-1.25	0.00051545	-1.24
ILMN_1721128	TOMM34	6.18E-05	-1.36	0.000298317	-1.34	0.00114211	-1.20	7.64E-05	-1.33
ILMN_3243302	C8orf30B	6.58E-05	-1.49	0.000640568	-1.33	0.00251699	-1.27	0.00121391	-1.32
ILMN_3235808	POM121C	6.61E-05	-1.44	0.000333337	-1.40	0.001405	-1.19	0.00026315	-1.26
ILMN_1656540	RUVBL1	0.00010606	-1.24	0.000761864	-1.17	0.00211344	-1.16	0.00091868	-1.18
ILMN_1721116	USP10	0.00010606	-1.35	0.000640568	-1.26	0.0004698	-1.26	0.00071989	-1.24
ILMN_1672728	KCTD5	0.00016479	-1.46	0.000298317	-1.51	1.77E-05	-1.51	0.0001877	-1.32
ILMN_2047112	RP11-529I10.4	0.00017064	-1.33	0.00053437	-1.28	0.00135057	-1.13	0.00071989	-1.15
ILMN_1776102	PSMD10	0.00026504	-1.22	0.000695377	-1.19	5.88E-05	-1.24	0.00027183	-1.19
ILMN_1713290	GLT8D1	0.00030614	-1.27	0.000843952	-1.23	0.00016916	-1.27	0.00125748	-1.18
ILMN_1728845	SMARCD1	0.00050023	-1.54	0.000298317	-1.76	1.49E-05	-1.42	5.36E-07	-1.79
ILMN_1682783	TUG1	0.00052675	-1.22	0.00053437	-1.23	0.00127383	-1.13	6.92E-05	-1.24
ILMN_1767219	POLR1C	0.00058616	-1.28	0.00144366	-1.24	0.00126828	-1.18	0.00141697	-1.18
ILMN_1708605	LOC652481	0.00099699	-1.21	0.000737322	-1.23	0.00094535	-1.18	0.0001579	-1.25
ILMN_1670028	LPIN2	0.00137847	-1.24	0.00103703	-1.25	4.25E-05	-1.35	5.36E-07	-1.79
ILMN_1693538	STK36	0.00155132	-1.34	0.000479142	-1.46	8.29E-05	-1.20	5.36E-07	-1.49
ILMN_1761969	DERL2	0.00170498	-1.38	0.000843952	-1.44	2.30E-05	-1.40	5.46E-06	-1.52
ILMN_2392674	PRR3	0.001756	-1.28	0.00103703	-1.31	0.00282174	-1.24	0.00192836	-1.26
ILMN_1803398	SRF	0.00309464	-1.31	0.000640568	-1.44	0.00019366	-1.26	1.15E-06	-1.67
ILMN_1668194	LMTK3	0.00454031	-1.10	0.00102618	-1.13	8.29E-05	-1.27	6.35E-05	-1.31
ILMN_3245458	SNORA61	0.00755162	-1.24	0.00118953	-1.35	0.00037278	-1.21	0.00012203	-1.27

* False discovery rate (FDR) adjusted p-value
