# Supplementary material for: LRH-1 drives colon cancer cell growth by repressing the expression of the CDKN1A gene in a p53-dependent manner
Source: Nucleic Acids Res. 2015 Sep 22;44(2):582–94. doi: 10.1093/nar/gkv948 (PMC4737183; doi:10.1093/nar/gkv948)
Supplement: SUPPLEMENTARY DATA [file supp_gkv948_nar-01236-x-2015-File016.pdf]

Supplementary Table 3. Genes up-regulated in HCT116 cells following LRH-1 siRNA

		HCT116 siLRH-1 #1 vs siLuc		HCT116 siLRH-1 #2 vs siLuc	
Probeset ID	*Gene Symbol*	*p-value**	*Fold Change*	*p-value**	*Fold Change*
ILMN_1749821	MED28	6.61E-08	2.20	1.42E-05	1.54
ILMN_1775708	SLC2A3	8.41E-08	3.51	3.90E-05	1.87
ILMN_1716053	AK2	2.65E-07	1.40	0.00040	1.12
ILMN_2225577	C5orf37	2.65E-07	1.43	0.00018	1.18
ILMN_3238435	SNORA12	2.65E-07	1.95	0.00018	1.37
ILMN_1697440	PRPF4	5.27E-07	1.33	0.00075	1.09
ILMN_1781285	DUSP1	5.33E-07	1.72	0.00021	1.30
ILMN_2401978	STAT3	9.67E-07	2.43	0.00021	1.60
ILMN_1663618	STAT3	9.67E-07	1.97	0.00035	1.36
ILMN_3237368	LOC644132	9.76E-07	1.73	0.00023	1.32
ILMN_1722894	ZNRD1	1.29E-06	1.44	0.00049	1.15
ILMN_1803279	TMED5	1.50E-06	1.93	0.00018	1.52
ILMN_1675939	IFNGR1	2.33E-06	1.57	0.00043	1.23
ILMN_2188264	CYR61	2.61E-06	1.51	0.00040	1.23
ILMN_1668619	KIAA1467	2.61E-06	1.63	0.00115	1.18
ILMN_2310589	DIABLO	3.45E-06	1.21	0.00113	1.07
ILMN_1799667	KIF4A	3.45E-06	1.34	0.00023	1.20
ILMN_1781097	UBXN4	3.94E-06	1.31	0.00066	1.12
ILMN_2133675	SGSH	4.43E-06	1.32	0.00150	1.10
ILMN_1766573	TERC	4.43E-06	2.13	0.00073	1.38
ILMN_1705116	C6orf85	5.13E-06	1.55	0.00021	1.36
ILMN_1807201	FAM104A	6.03E-06	1.50	0.00034	1.29
ILMN_1803476	KCTD20	6.03E-06	1.55	0.00068	1.22
ILMN_2224143	MCM3	6.03E-06	1.19	0.00020	1.14
ILMN_2054392	PPIL1	6.03E-06	1.29	0.00040	1.16
ILMN_1732410	SLC16A9	6.25E-06	1.38	0.00144	1.13
ILMN_2410986	STAT3	6.25E-06	1.95	0.00052	1.40
ILMN_2412761	MAFG	6.78E-06	1.50	0.00127	1.17
ILMN_3241373	SCARNA18	7.83E-06	1.69	0.00115	1.24
ILMN_2325347	B3GALNT1	8.48E-06	1.80	0.00022	1.58
ILMN_1725485	RGS17	9.45E-06	1.23	0.00076	1.10
ILMN_1708416	ARL6IP1	1.02E-05	1.55	0.00040	1.32
ILMN_1762531	FGF9	1.07E-05	1.44	0.00035	1.29
ILMN_2096322	ADIPOR1	1.11E-05	1.39	0.00058	1.20
ILMN_1736054	SUB1	1.28E-05	1.31	0.00069	1.15
ILMN_1761259	EXT2	1.28E-05	1.22	0.00108	1.10
ILMN_2163306	FAM120A	1.28E-05	1.23	0.00115	1.10
ILMN_1764177	JARID2	1.28E-05	1.42	0.00041	1.26
ILMN_2348975	NASP	1.28E-05	1.25	0.00063	1.13
ILMN_1749011	NECAP2	1.28E-05	1.15	0.00061	1.08
ILMN_1655990	CDK5RAP2	1.39E-05	1.28	0.00043	1.17
ILMN_2330371	TATDN3	1.56E-05	1.40	0.00049	1.23
ILMN_2397231	USP16	1.57E-05	1.28	0.00115	1.12
ILMN_1683096	ASB1	1.60E-05	1.31	0.00103	1.15
ILMN_2231911	AUH	1.60E-05	1.25	0.00068	1.14
ILMN_1685824	B4GALT5	1.60E-05	1.58	0.00022	1.49
ILMN_1707339	BTG3	1.60E-05	1.63	0.00030	1.48
ILMN_1676322	C1orf172	1.60E-05	1.25	0.00115	1.11
ILMN_1712803	CCNB1	1.60E-05	1.44	0.00075	1.22
ILMN_1668507	DDAH1	1.60E-05	1.45	0.00138	1.18
ILMN_3308335	RNU6-1	1.60E-05	1.34	0.00103	1.16
ILMN_2324998	NGDN	1.93E-05	1.36	0.00077	1.19
ILMN_1721713	EXOSC9	2.00E-05	1.15	0.00037	1.11
ILMN_1777106	LRRC57	2.40E-05	1.43	0.00041	1.30

ILMN_1679881	WRN	2.40E-05	1.22	0.00049	1.15
ILMN_1736901	ZDHHC23	2.40E-05	1.26	0.00034	1.21
ILMN_1875123	HS.572219	2.40E-05	1.46	0.00097	1.23
ILMN_2124471	SLC36A1	2.56E-05	1.45	0.00051	1.28
ILMN_2415911	ENOX2	2.62E-05	1.35	0.00063	1.21
ILMN_1707748	PIM3	2.93E-05	1.26	0.00044	1.18
ILMN_1683127	ZNF281	2.98E-05	1.48	0.00111	1.24
ILMN_2383934	ITGB1	3.09E-05	1.56	0.00037	1.44
ILMN_1657697	SAR1A	3.13E-05	1.47	0.00023	1.45
ILMN_1736510	FOXN2	3.39E-05	1.41	0.00037	1.33
ILMN_1683664	LOC650369	3.41E-05	1.26	0.00079	1.15
ILMN_1776038	UBXN2A	3.41E-05	1.35	0.00060	1.22
ILMN_1750181	TESC	3.41E-05	1.19	0.00047	1.14
ILMN_1806818	MCM3	3.92E-05	1.32	0.00099	1.18
ILMN_1810436	DNAJC27	3.93E-05	1.30	0.00030	1.28
ILMN_1801822	C18orf25	3.96E-05	1.39	0.00049	1.27
ILMN_1652907	CDC5L	4.38E-05	1.23	0.00068	1.15
ILMN_1741171	TM2D2	4.38E-05	1.59	0.00068	1.37
ILMN_1785618	SMTN	4.55E-05	1.50	0.00040	1.41
ILMN_1701681	SEC11C	4.66E-05	1.30	0.00065	1.20
ILMN_1714820	ITGB1	4.79E-05	1.52	0.00040	1.42
ILMN_1711799	C9orf40	5.07E-05	1.22	0.00050	1.16
ILMN_1770803	BNIP2	5.16E-05	1.29	0.00040	1.24
ILMN_1761961	CCDC90A	5.16E-05	1.15	0.00049	1.12
ILMN_1801257	CENPA	5.16E-05	1.24	0.00150	1.12
ILMN_1669523	FOS	5.16E-05	1.35	0.00116	1.19
ILMN_1652434	MTHFD2L	5.16E-05	1.33	0.00105	1.19
ILMN_1669931	TM9SF3	5.17E-05	1.37	0.00073	1.24
ILMN_2403965	TXNDC5	5.17E-05	1.22	0.00103	1.13
ILMN_2354478	CYFIP2	5.42E-05	1.25	0.00035	1.23
ILMN_1801939	CCNB2	5.74E-05	1.29	0.00041	1.24
ILMN_1687074	CDC14B	5.74E-05	1.29	0.00144	1.15
ILMN_1837935	TNPO1	5.75E-05	1.34	0.00073	1.22
ILMN_1802669	PPP3CB	6.36E-05	1.40	0.00063	1.29
ILMN_1698019	LGMN	6.91E-05	1.21	0.00151	1.11
ILMN_2390338	UBE2E3	6.97E-05	1.30	0.00099	1.19
ILMN_2293992	RAB28	7.16E-05	1.32	0.00099	1.20
ILMN_1689336	HOXA10	7.70E-05	1.64	0.00035	1.64
ILMN_1731640	MGC39900	8.00E-05	1.33	0.00048	1.27
ILMN_1737343	FNIP1	8.24E-05	1.32	0.00063	1.24
ILMN_1715175	MET	8.34E-05	1.39	0.00070	1.28
ILMN_1740216	ERCC3	8.45E-05	1.27	0.00040	1.25
ILMN_1777061	ZSWIM6	8.74E-05	1.22	0.00111	1.14
ILMN_1662318	CCDC59	8.81E-05	1.15	0.00061	1.12
ILMN_1659843	DNAJC3	9.36E-05	1.26	0.00103	1.17
ILMN_2099301	UNC84B	9.59E-05	1.45	0.00129	1.26
ILMN_3211857	LOC648822	9.83E-05	1.37	0.00063	1.28
ILMN_1776052	LOC148915	9.91E-05	1.30	0.00040	1.29
ILMN_1746856	RAB21	9.99E-05	1.39	0.00126	1.24
ILMN_2389114	FIGNL1	0.00010	1.32	0.00102	1.21
ILMN_1677765	LRP8	0.00011	1.32	0.00049	1.28
ILMN_1654488	UTX	0.00011	1.23	0.00121	1.15
ILMN_3227263	SLC22A23	0.00011	1.24	0.00115	1.15
ILMN_2187718	COX17	0.00011	1.23	0.00144	1.14
ILMN_1778240	GFOD1	0.00012	1.16	0.00044	1.15
ILMN_1685661	RRP15	0.00012	1.18	0.00150	1.11
ILMN_1779813	FAM96B	0.00012	1.37	0.00115	1.24
ILMN_3307700	SPCS3	0.00013	1.16	0.00040	1.16

ILMN_1655644	ZNF804A	0.00013	1.25	0.00147	1.15
ILMN_1694325	NFIX	0.00013	1.34	0.00101	1.24
ILMN_1681737	TMSB15A	0.00013	1.34	0.00044	1.33
ILMN_1720850	BAZ2B	0.00014	1.18	0.00115	1.12
ILMN_3193306	C14orf109	0.00014	1.24	0.00155	1.15
ILMN_1788108	TXNDC5	0.00015	1.34	0.00065	1.28
ILMN_2320336	CLK3	0.00015	1.26	0.00143	1.17
ILMN_1808768	ROCK1	0.00015	1.37	0.00040	1.39
ILMN_1676629	INSIG2	0.00015	1.40	0.00086	1.30
ILMN_1682110	HOXA10	0.00016	1.26	0.00040	1.28
ILMN_2062381	LCOR	0.00016	1.60	0.00049	1.56
ILMN_1652357	PDHX	0.00016	1.27	0.00099	1.20
ILMN_2089656	C1orf107	0.00017	1.27	0.00055	1.25
ILMN_1742224	SLTM	0.00017	1.19	0.00043	1.19
ILMN_1675797	EPDR1	0.00017	1.64	0.00043	1.66
ILMN_1784602	CDKN1A	0.00018	1.33	0.00040	1.37
ILMN_2400500	LASS2	0.00018	1.42	0.00083	1.33
ILMN_1760849	NETO2	0.00018	1.43	0.00049	1.41
ILMN_1814726	SCARB2	0.00018	1.23	0.00147	1.15
ILMN_1760683	SFRS9	0.00018	1.21	0.00142	1.14
ILMN_1737283	MGC39900	0.00019	1.23	0.00044	1.23
ILMN_1759117	XK	0.00019	1.34	0.00048	1.33
ILMN_1694385	YWHAB	0.00019	1.22	0.00053	1.21
ILMN_1677466	DUSP6	0.00020	1.25	0.00132	1.17
ILMN_2189222	KLHL8	0.00020	1.22	0.00063	1.19
ILMN_3279219	LOC100132457	0.00022	1.17	0.00146	1.12
ILMN_1743130	PTGFRN	0.00022	1.29	0.00120	1.21
ILMN_2415979	KIAA1751	0.00022	1.24	0.00075	1.21
ILMN_2198893	LOC407835	0.00022	1.22	0.00116	1.16
ILMN_1691143	RAB18	0.00022	1.33	0.00068	1.29
ILMN_2058141	HMGN2	0.00023	1.28	0.00049	1.28
ILMN_1798108	C6orf211	0.00023	1.20	0.00115	1.15
ILMN_2354140	NAT5	0.00024	1.19	0.00132	1.14
ILMN_1800912	ZFP106	0.00025	1.27	0.00097	1.22
ILMN_1669553	UBE2E3	0.00026	1.26	0.00144	1.18
ILMN_2169439	ITGAV	0.00028	1.28	0.00124	1.21
ILMN_1711919	SCYL2	0.00031	1.36	0.00136	1.27
ILMN_2167922	TRMT5	0.00031	1.26	0.00065	1.25
ILMN_1802257	PCTP	0.00032	1.25	0.00113	1.20
ILMN_1682930	SIPA1	0.00034	1.20	0.00146	1.15
ILMN_1753639	MTAP	0.00035	1.14	0.00103	1.12
ILMN_1755792	STK38L	0.00035	1.26	0.00088	1.23
ILMN_1728001	SFRS12IP1	0.00035	1.18	0.00099	1.15
ILMN_1711139	VPS37A	0.00035	1.26	0.00130	1.20
ILMN_2373495	H2AFY	0.00037	1.22	0.00049	1.24
ILMN_1724495	SESTD1	0.00038	1.38	0.00077	1.35
ILMN_2194467	SGCB	0.00038	1.20	0.00043	1.23
ILMN_2279961	LAMP2	0.00039	1.25	0.00076	1.23
ILMN_1750880	AMN1	0.00040	1.13	0.00149	1.10
ILMN_1712305	CYBRD1	0.00040	1.26	0.00108	1.22
ILMN_1727194	CALU	0.00041	1.24	0.00059	1.25
ILMN_1728225	KIAA1524	0.00042	1.23	0.00041	1.28
ILMN_1794392	DDX3X	0.00043	1.38	0.00051	1.42
ILMN_1742541	ZNF518A	0.00044	1.21	0.00073	1.20
ILMN_1765332	TIMM10	0.00045	1.18	0.00088	1.16
ILMN_2223903	PPIC	0.00046	1.18	0.00076	1.18
ILMN_3199920	LOC100132352	0.00047	1.20	0.00144	1.16
ILMN_1714167	CYB5A	0.00051	1.21	0.00150	1.16

ILMN_1737025	PLCL2	0.00051	1.19	0.00088	1.18
ILMN_1753342	SAT1	0.00051	1.28	0.00049	1.33
ILMN_2161832	VPS37A	0.00051	1.25	0.00149	1.20
ILMN_2103841	AIP	0.00053	1.22	0.00115	1.19
ILMN_1673026	CHCHD3	0.00053	1.38	0.00113	1.33
ILMN_1738263	PIGU	0.00053	1.23	0.00127	1.19
ILMN_1726786	TNRC6B	0.00054	1.47	0.00053	1.54
ILMN_1714759	CNIH4	0.00054	1.25	0.00150	1.20
ILMN_2285817	FAM89A	0.00054	1.20	0.00101	1.18
ILMN_1741219	RRAGB	0.00054	1.27	0.00133	1.22
ILMN_1654141	ZDHHC20	0.00061	1.19	0.00139	1.16
ILMN_1787511	THUMPD2	0.00061	1.22	0.00144	1.18
ILMN_1714730	UBE2C	0.00064	1.19	0.00137	1.17
ILMN_1693669	WDR79	0.00067	1.30	0.00122	1.27
ILMN_1703279	CXorf57	0.00067	1.24	0.00045	1.31
ILMN_1683044	PPP1R2	0.00067	1.23	0.00099	1.22
ILMN_2154836	BTG3	0.00068	1.36	0.00075	1.38
ILMN_1786720	PROM1	0.00069	1.21	0.00115	1.19
ILMN_1810962	PTPRK	0.00070	1.24	0.00150	1.20
ILMN_2410864	RAB28	0.00071	1.24	0.00111	1.23
ILMN_1811367	MAT2B	0.00072	1.29	0.00116	1.26
ILMN_1735594	CDC42SE2	0.00080	1.14	0.00139	1.13
ILMN_2054297	PTGS2	0.00080	1.27	0.00113	1.26
ILMN_1731374	CPE	0.00083	1.22	0.00099	1.22
ILMN_1747020	SGK3	0.00085	1.15	0.00113	1.14
ILMN_1783910	TRAF6	0.00085	1.17	0.00115	1.17
ILMN_1778152	FIGNL1	0.00086	1.24	0.00115	1.23
ILMN_1667839	UBR7	0.00087	1.25	0.00108	1.25
ILMN_2043265	ZFP106	0.00089	1.18	0.00150	1.16
ILMN_1655165	RNF138	0.00090	1.20	0.00148	1.18
ILMN_1779147	ENC1	0.00094	1.18	0.00136	1.16
ILMN_2367010	GPR126	0.00094	1.25	0.00082	1.28
ILMN_1740407	CHSY3	0.00094	1.19	0.00073	1.22
ILMN_1723092	CRB3	0.00095	1.31	0.00066	1.37
ILMN_2162989	TMEM189	0.00096	1.27	0.00150	1.24
ILMN_2134555	KCTD3	0.00101	1.15	0.00128	1.14
ILMN_1800220	KCTD3	0.00105	1.23	0.00099	1.24
ILMN_3237946	LOC100134134	0.00105	1.26	0.00147	1.24
ILMN_1897741	HS.137971	0.00105	1.21	0.00097	1.22
ILMN_1667417	RAB23	0.00109	1.19	0.00049	1.26
ILMN_1699489	TUBB6	0.00111	1.23	0.00075	1.26
ILMN_1869087	HS.40289	0.00127	1.35	0.00075	1.42
ILMN_3233388	RELL1	0.00138	1.32	0.00061	1.43
ILMN_1806502	ZNF165	0.00151	1.22	0.00099	1.26
ILMN_1768197	ROD1	0.00162	1.23	0.00048	1.34
ILMN_3290100	LOC645157	0.00187	1.16	0.00148	1.16
ILMN_3302139	LOC729687	0.00190	1.20	0.00111	1.23
ILMN_2079786	NUAK1	0.00193	1.25	0.00147	1.26
ILMN_1785765	TM9SF2	0.00195	1.14	0.00147	1.15
ILMN_2077550	RACGAP1	0.00204	1.24	0.00051	1.35
ILMN_1689274	NIPA1	0.00205	1.25	0.00101	1.30
ILMN_2136455	C3orf64	0.00229	1.29	0.00115	1.34
ILMN_2126706	LMNB1	0.00231	1.20	0.00075	1.26
ILMN_1745365	PKNOX1	0.00231	1.15	0.00073	1.21
ILMN_1698533	IDH3A	0.00237	1.17	0.00150	1.18
ILMN_1677404	RAP2A	0.00238	1.27	0.00142	1.29
ILMN_3244521	LOC283267	0.00240	1.23	0.00150	1.25
ILMN_1734229	SPPL2A	0.00240	1.19	0.00115	1.23

ILMN_2349831	DICER1	0.00251	1.25	0.00102	1.31
ILMN_1700024	UST	0.00255	1.38	0.00053	1.60
ILMN_1691616	LOC727935	0.00263	1.09	0.00115	1.11
ILMN_2047599	TMEM50B	0.00266	1.10	0.00057	1.14
ILMN_1797372	C3orf58	0.00269	1.26	0.00103	1.33
ILMN_1698677	C4orf27	0.00278	1.08	0.00116	1.10
ILMN_1733937	MMD	0.00289	1.20	0.00097	1.26
ILMN_1686097	TOP2A	0.00293	1.16	0.00115	1.19
ILMN_1713759	UBE2J1	0.00294	1.21	0.00073	1.30
ILMN_2238928	RAD51C	0.00296	1.13	0.00127	1.15
ILMN_1751276	BDNF	0.00299	1.18	0.00070	1.26
ILMN_1772316	UNC84A	0.00306	1.20	0.00108	1.26
ILMN_1673673	PBK	0.00322	1.25	0.00060	1.40
ILMN_2219618	LOC90586	0.00323	1.12	0.00097	1.16
ILMN_2233539	SLC39A8	0.00340	1.18	0.00144	1.21
ILMN_1728540	FUNDC1	0.00355	1.10	0.00108	1.13
ILMN_3236346	LOC100132901	0.00355	1.14	0.00144	1.16
ILMN_1730575	GCLC	0.00371	1.15	0.00069	1.23
ILMN_1804351	FZD7	0.00436	1.16	0.00115	1.21
ILMN_1651433	DCK	0.00441	1.17	0.00106	1.23
ILMN_1674344	PARD6G	0.00473	1.22	0.00115	1.30
ILMN_1664855	PPP1R14C	0.00487	1.36	0.00057	1.65
ILMN_2365881	ATG16L1	0.00527	1.16	0.00115	1.22
ILMN_2038775	TUBB2A	0.00531	1.12	0.00127	1.16
ILMN_2093027	MYO1B	0.00568	1.14	0.00115	1.20
ILMN_3242038	GPX8	0.00576	1.15	0.00073	1.25
ILMN_2068435	ZNF700	0.00582	1.14	0.00075	1.23
ILMN_1710428	CDC2	0.00669	1.29	0.00151	1.39
ILMN_1660723	RDH13	0.00707	1.14	0.00113	1.21
ILMN_2224907	C4orf34	0.00717	1.13	0.00065	1.23
ILMN_1788019	LAMA2	0.00744	1.20	0.00103	1.31
ILMN_3251317	RER1	0.00757	1.12	0.00138	1.17
ILMN_2072541	RAB11FIP2	0.00792	1.19	0.00126	1.28
ILMN_2199947	REV3L	0.00834	1.12	0.00099	1.19
ILMN_1666739	RBM15	0.00840	1.13	0.00144	1.18
ILMN_1801403	DCUN1D4	0.00852	1.12	0.00150	1.16

* False discovery rate (FDR) adjusted p-value
