# Supplementary material for: LRH-1 drives colon cancer cell growth by repressing the expression of the CDKN1A gene in a p53-dependent manner
Source: Nucleic Acids Res. 2015 Sep 22;44(2):582–94. doi: 10.1093/nar/gkv948 (PMC4737183; doi:10.1093/nar/gkv948)
Supplement: SUPPLEMENTARY DATA [file supp_gkv948_nar-01236-x-2015-File017.pdf]

Supplementary Table 4. Genes down-regulated in HCT116 cells following LRH-1 siRNA

		**HCT116 siLRH-1 #1 vs siLuc**		**HCT116 siLRH-1 #2 vs siLuc**	
Probeset ID	*Gene Symbol*	*p-value**	*Fold Change*	*p-value**	*Fold Change*
ILMN_2111918	C14orf135	1.33E-07	-1.56	0.00013	-1.20
ILMN_1687315	RXRA	3.75E-07	-1.64	0.00013	-1.27
ILMN_1701514	TRAF3IP2	6.09E-07	-1.28	0.00013	-1.14
ILMN_1675709	ARFGAP1	7.03E-07	-1.30	0.00033	-1.12
ILMN_1695792	CUL4A	7.03E-07	-1.62	0.00013	-1.30
ILMN_1757882	PPP1R16A	7.03E-07	-1.84	0.00020	-1.31
ILMN_1732049	DPM2	1.15E-06	-2.36	0.00152	-1.26
ILMN_1771841	FOSL1	1.15E-06	-1.77	0.00014	-1.37
ILMN_2311537	HMGA1	1.37E-06	-1.63	0.00071	-1.20
ILMN_1740395	RAVER1	1.37E-06	-1.92	0.00043	-1.33
ILMN_1662852	IQCK	1.50E-06	-1.46	0.00016	-1.24
ILMN_2399497	RFXANK	1.50E-06	-1.47	0.00016	-1.25
ILMN_3236244	C1orf174	1.71E-06	-2.11	0.00102	-1.29
ILMN_3233930	LOC390557	2.27E-06	-1.23	0.00033	-1.12
ILMN_1794692	DNMT3B	2.77E-06	-1.48	0.00016	-1.29
ILMN_2210729	STYXL1	2.77E-06	-1.27	0.00013	-1.20
ILMN_2328972	DNMT3B	3.08E-06	-1.91	0.00014	-1.56
ILMN_1707137	C17orf97	3.44E-06	-1.67	0.00076	-1.24
ILMN_1651358	HBE1	3.74E-06	-1.80	0.00102	-1.26
ILMN_1695311	HLA-DMA	4.28E-06	-1.34	0.00098	-1.13
ILMN_1784985	PRRT3	4.88E-06	-1.53	0.00043	-1.27
ILMN_1815878	C11orf59	5.15E-06	-1.30	0.00067	-1.14
ILMN_2331163	CUL4A	5.15E-06	-1.43	0.00043	-1.23
ILMN_1794501	HAS3	5.15E-06	-2.36	0.00013	-2.04
ILMN_1748625	TCEAL4	5.15E-06	-1.24	0.00043	-1.13
ILMN_2212354	WDR46	5.73E-06	-1.37	0.00076	-1.16
ILMN_1760718	ZMIZ2	6.51E-06	-1.69	0.00100	-1.26
ILMN_2179726	C16orf93	6.91E-06	-1.56	0.00098	-1.22
ILMN_1681972	TMEM69	7.94E-06	-1.94	0.00095	-1.36
ILMN_1797181	LOC93622	9.00E-06	-1.40	0.00095	-1.17
ILMN_1751984	PRKAG1	9.00E-06	-1.25	0.00033	-1.17
ILMN_1651735	TGOLN2	9.00E-06	-1.23	0.00057	-1.12
ILMN_1804117	FAM89B	1.00E-05	-1.54	0.00079	-1.24
ILMN_1651832	EHD1	1.03E-05	-1.41	0.00097	-1.18
ILMN_1685124	TCTN1	1.03E-05	-1.40	0.00079	-1.19
ILMN_1799579	CCDC51	1.07E-05	-1.32	0.00128	-1.13
ILMN_1758831	RNF31	1.07E-05	-1.44	0.00076	-1.21
ILMN_1757437	UMPS	1.09E-05	-1.33	0.00084	-1.16
ILMN_1668760	HAS3	1.14E-05	-1.52	0.00093	-1.24
ILMN_1759232	IRS1	1.20E-05	-1.29	0.00045	-1.18
ILMN_1660067	LOC728285	1.20E-05	-1.55	0.00057	-1.31
ILMN_1739813	HYAL1	1.36E-05	-1.60	0.00014	-1.54
ILMN_3238785	SNHG9	1.38E-05	-1.33	0.00095	-1.16
ILMN_1723709	C9orf116	1.39E-05	-1.32	0.00043	-1.21
ILMN_1657475	GALT	1.41E-05	-1.25	0.00076	-1.13
ILMN_2185339	LRTOMT	1.43E-05	-1.36	0.00136	-1.15
ILMN_1724941	CDCP1	1.55E-05	-1.33	0.00057	-1.20
ILMN_1655635	METTL3	1.69E-05	-1.62	0.00028	-1.49
ILMN_1814022	NR1H3	1.69E-05	-1.25	0.00057	-1.16
ILMN_3290340	LOC100132032	1.71E-05	-1.21	0.00108	-1.10
ILMN_2355042	CLUAP1	1.90E-05	-1.30	0.00056	-1.19
ILMN_1687092	KBTBD4	2.29E-05	-1.34	0.00055	-1.23
ILMN_1763568	ZDHHC16	2.29E-05	-1.26	0.00102	-1.14
ILMN_1738027	BRCA1	2.37E-05	-1.39	0.00106	-1.19

ILMN_1667257	SDHB	2.37E-05	-1.31	0.00076	-1.18
ILMN_2325112	C22orf40	2.41E-05	-1.33	0.00146	-1.15
ILMN_1686664	MT2A	2.41E-05	-1.51	0.00055	-1.34
ILMN_1669394	EI24	2.45E-05	-1.24	0.00071	-1.15
ILMN_2348146	ERCC8	2.76E-05	-1.13	0.00071	-1.08
ILMN_1764927	CDC42EP1	2.76E-05	-1.38	0.00071	-1.24
ILMN_1735199	CIAPIN1	3.36E-05	-1.25	0.00154	-1.12
ILMN_1813625	TRIM25	3.39E-05	-1.32	0.00071	-1.21
ILMN_1713732	ABL1	3.50E-05	-1.32	0.00043	-1.26
ILMN_1690982	DDT	3.50E-05	-1.28	0.00073	-1.18
ILMN_1876266	GJA3	3.50E-05	-1.37	0.00071	-1.24
ILMN_1691425	LDOC1L	3.50E-05	-1.43	0.00150	-1.21
ILMN_3238751	PMS2L4	3.61E-05	-1.23	0.00115	-1.12
ILMN_3206827	LOC100131737	3.69E-05	-1.49	0.00095	-1.28
ILMN_1755711	C17orf68	3.87E-05	-1.48	0.00128	-1.25
ILMN_1752406	LOC642282	4.50E-05	-1.44	0.00102	-1.25
ILMN_1704024	TMEM160	4.52E-05	-1.33	0.00112	-1.18
ILMN_1737475	ABHD11	4.52E-05	-1.18	0.00149	-1.09
ILMN_2408039	EEF1D	4.68E-05	-1.14	0.00115	-1.08
ILMN_1669362	IGFBP6	4.68E-05	-1.37	0.00136	-1.20
ILMN_1678934	POLR1E	5.00E-05	-1.26	0.00079	-1.17
ILMN_1780987	RFXANK	5.11E-05	-1.39	0.00033	-1.38
ILMN_2365544	NHP2	6.68E-05	-1.20	0.00096	-1.13
ILMN_2363489	BRE	6.71E-05	-1.36	0.00106	-1.22
ILMN_1771697	VRK3	6.71E-05	-1.50	0.00150	-1.27
ILMN_1722239	TIMM8A	7.02E-05	-1.29	0.00042	-1.27
ILMN_1796968	INTS5	7.05E-05	-1.28	0.00043	-1.24
ILMN_1759766	CTXN1	7.70E-05	-1.27	0.00115	-1.17
ILMN_1803945	HCP5	7.70E-05	-1.23	0.00106	-1.15
ILMN_3237584	LOC100133489	7.70E-05	-1.27	0.00128	-1.16
ILMN_3244323	LOC148413	8.02E-05	-1.19	0.00152	-1.11
ILMN_1750273	RPL23AP7	8.02E-05	-1.40	0.00076	-1.29
ILMN_1744308	DHX33	8.57E-05	-1.38	0.00043	-1.36
ILMN_1746232	KITLG	8.57E-05	-1.38	0.00071	-1.29
ILMN_1774974	CLUAP1	8.91E-05	-1.24	0.00102	-1.16
ILMN_1701621	SCO2	9.50E-05	-1.31	0.00092	-1.21
ILMN_1713901	KDELR3	0.00012	-1.33	0.00136	-1.21
ILMN_1701134	PTEN	0.00012	-1.17	0.00071	-1.14
ILMN_1798177	CHURC1	0.00014	-1.22	0.00043	-1.22
ILMN_1718309	COX15	0.00014	-1.23	0.00079	-1.18
ILMN_2323801	MOCS1	0.00014	-1.32	0.00071	-1.28
ILMN_2048982	ZBTB25	0.00014	-1.17	0.00102	-1.12
ILMN_1722820	KDELR3	0.00014	-1.44	0.00042	-1.47
ILMN_1710598	SKI	0.00014	-1.18	0.00033	-1.20
ILMN_1796210	PPRC1	0.00016	-1.38	0.00033	-1.44
ILMN_1774083	TRIAP1	0.00016	-1.25	0.00082	-1.19
ILMN_1695946	TRNP1	0.00016	-1.29	0.00088	-1.22
ILMN_1797933	MRPL17	0.00017	-1.22	0.00095	-1.17
ILMN_1653367	TAF12	0.00017	-1.37	0.00057	-1.36
ILMN_1730940	KLHDC3	0.00017	-1.23	0.00146	-1.15
ILMN_2043615	C17orf90	0.00020	-1.17	0.00155	-1.11
ILMN_1714861	CD68	0.00020	-1.20	0.00136	-1.14
ILMN_1754126	SH2D5	0.00023	-1.25	0.00117	-1.18
ILMN_2345837	CLTA	0.00023	-1.16	0.00043	-1.18
ILMN_1762071	C17orf80	0.00025	-1.39	0.00108	-1.30
ILMN_1739345	C11orf48	0.00026	-1.26	0.00057	-1.26
ILMN_1652331	KCTD5	0.00035	-1.17	0.00076	-1.16
ILMN_1738529	BCS1L	0.00037	-1.20	0.00139	-1.15

ILMN_1772888	LOC645688	0.00037	-1.20	0.00105	-1.16
ILMN_1651886	CWF19L1	0.00042	-1.23	0.00145	-1.18
ILMN_3223126	TYMP	0.00042	-1.24	0.00105	-1.21
ILMN_1657111	C14orf78	0.00042	-1.26	0.00082	-1.24
ILMN_3248758	LOC728934	0.00045	-1.23	0.00079	-1.22
ILMN_2201533	C17orf61	0.00045	-1.24	0.00135	-1.19
ILMN_1691156	MT1A	0.00046	-1.22	0.00071	-1.23
ILMN_1687430	EIF2B4	0.00048	-1.11	0.00149	-1.09
ILMN_2347068	MKNK2	0.00048	-1.37	0.00071	-1.39
ILMN_1752478	DHRS3	0.00051	-1.19	0.00096	-1.18
ILMN_1746686	POLR1C	0.00054	-1.25	0.00102	-1.22
ILMN_3181420	HMGXB4	0.00056	-1.24	0.00100	-1.22
ILMN_3215008	LOC644153	0.00056	-1.14	0.00139	-1.11
ILMN_3242091	NCRNA00094	0.00057	-1.32	0.00071	-1.34
ILMN_2381121	UQCC	0.00062	-1.23	0.00080	-1.23
ILMN_1806122	CHD8	0.00065	-1.30	0.00149	-1.25
ILMN_3229570	LOC729500	0.00068	-1.17	0.00106	-1.15
ILMN_1698934	CMTM7	0.00071	-1.19	0.00071	-1.22
ILMN_1815158	GPS2	0.00075	-1.15	0.00114	-1.14
ILMN_1745570	KLK7	0.00080	-1.25	0.00098	-1.25
ILMN_2113807	MYL2	0.00080	-1.14	0.00076	-1.15
ILMN_1692785	KLHL21	0.00086	-1.16	0.00136	-1.15
ILMN_1740487	CMTM7	0.00088	-1.21	0.00071	-1.24
ILMN_1736340	ANGEL2	0.00089	-1.28	0.00071	-1.32
ILMN_1709032	FYCO1	0.00089	-1.39	0.00050	-1.55
ILMN_1785095	ATP6V0E2	0.00098	-1.25	0.00148	-1.23
ILMN_1815519	EPN2	0.00098	-1.31	0.00076	-1.36
ILMN_1795026	FAM189B	0.00100	-1.27	0.00076	-1.31
ILMN_1862018	ATXN7L3	0.00103	-1.25	0.00056	-1.34
ILMN_1778032	SURF6	0.00103	-1.12	0.00076	-1.13
ILMN_1736814	CLNS1A	0.00104	-1.21	0.00095	-1.22
ILMN_1820244	HS.535360	0.00106	-1.24	0.00071	-1.29
ILMN_1788347	KIAA1737	0.00112	-1.26	0.00076	-1.31
ILMN_2168347	EPOR	0.00117	-1.23	0.00079	-1.26
ILMN_1651506	NCOA6IP	0.00120	-1.24	0.00095	-1.26
ILMN_1724479	NR2C2	0.00120	-1.12	0.00128	-1.12
ILMN_1660292	MRPS21	0.00123	-1.22	0.00071	-1.28
ILMN_1680618	MYC	0.00127	-1.26	0.00091	-1.29
ILMN_2385220	DFFA	0.00131	-1.18	0.00095	-1.20
ILMN_1688725	UTP14A	0.00158	-1.20	0.00082	-1.24
ILMN_2158003	KIAA1683	0.00159	-1.13	0.00106	-1.14
ILMN_1794707	ATHL1	0.00167	-1.15	0.00093	-1.18
ILMN_1776490	C17orf53	0.00167	-1.18	0.00149	-1.18
ILMN_1743049	PWP1	0.00168	-1.10	0.00071	-1.13
ILMN_2051867	PTCD2	0.00168	-1.16	0.00071	-1.21
ILMN_2063168	MALL	0.00184	-1.19	0.00056	-1.29
ILMN_1753819	RFFL	0.00189	-1.29	0.00149	-1.29
ILMN_1657002	OR51B5	0.00203	-1.24	0.00057	-1.36
ILMN_1693352	MRPL20	0.00207	-1.14	0.00111	-1.16
ILMN_1733757	LOC374395	0.00221	-1.18	0.00079	-1.24
ILMN_3248076	LOC100131266	0.00227	-1.18	0.00149	-1.19
ILMN_2097858	KIAA1737	0.00282	-1.26	0.00149	-1.29
ILMN_1691090	MPV17	0.00284	-1.19	0.00148	-1.21
ILMN_1808500	CEP68	0.00304	-1.17	0.00095	-1.22
ILMN_2164081	KLHL12	0.00337	-1.23	0.00149	-1.27
ILMN_2110908	MYC	0.00348	-1.17	0.00113	-1.21
ILMN_1796461	PRSS8	0.00348	-1.16	0.00095	-1.21
ILMN_1664861	ID1	0.00360	-1.23	0.00120	-1.28

ILMN_1662848	TXNDC15	0.00367	-1.17	0.00113	-1.22
ILMN_1661220	TRIM16	0.00381	-1.18	0.00146	-1.21
ILMN_2340877	MEN1	0.00423	-1.23	0.00076	-1.35
ILMN_1653797	C6orf62	0.00484	-1.22	0.00136	-1.28
ILMN_1712887	SLC10A3	0.00505	-1.25	0.00128	-1.33
ILMN_2227533	ABHD14B	0.00510	-1.20	0.00102	-1.28
ILMN_1755974	ALDOC	0.00510	-1.16	0.00113	-1.22
ILMN_1748908	PROSC	0.00527	-1.07	0.00071	-1.11
ILMN_1746561	BCL2L2	0.00538	-1.13	0.00113	-1.18
ILMN_2366998	CHTF8	0.00561	-1.14	0.00079	-1.23
ILMN_1734762	OPA3	0.00561	-1.16	0.00071	-1.27
ILMN_3251629	EIF4A1	0.00600	-1.20	0.00128	-1.27
ILMN_1673450	DDN	0.00672	-1.16	0.00136	-1.22
ILMN_1811102	LRSAM1	0.00707	-1.22	0.00057	-1.43
ILMN_2307032	OSBPL5	0.00767	-1.14	0.00102	-1.21

* False discovery rate (FDR) adjusted p-value
