# Supplementary material for: LRH-1 drives colon cancer cell growth by repressing the expression of the CDKN1A gene in a p53-dependent manner
Source: Nucleic Acids Res. 2015 Sep 22;44(2):582–94. doi: 10.1093/nar/gkv948 (PMC4737183; doi:10.1093/nar/gkv948)
Supplement: SUPPLEMENTARY DATA [file supp_gkv948_nar-01236-x-2015-File018.pdf]

Supplementary Table 5. Genes up-regulated in HT29 cells following LRH-1 siRNA

		HT29 siLRH-1 #1 vs siLuc		HT29 siLRH-1 #2 vs siLuc	
Probeset ID	*Gene Symbol*	*p-value**	*Fold Change*	*p-value**	*Fold Change*
ILMN_1754489	FBXL20	2.68E-05	1.31	6.15E-05	1.20
ILMN_1717099	DSCR3	5.20E-05	1.49	0.00022	1.28
ILMN_1713505	NPC1	5.20E-05	1.27	1.85E-05	1.33
ILMN_1741371	TMEM8	5.20E-05	1.52	4.62E-05	1.52
ILMN_1741264	MRPS33	7.97E-05	1.22	0.00016	1.15
ILMN_1669502	E2F3	0.00011	1.22	0.00020	1.15
ILMN_1699598	AP2M1	0.00014	1.19	0.00080	1.10
ILMN_2243308	ACVR1B	0.00014	1.29	0.00035	1.21
ILMN_3247802	BAT2L	0.00014	1.16	0.00070	1.10
ILMN_3244065	C9orf69	0.00014	1.23	0.00011	1.22
ILMN_2261973	DPH3	0.00014	1.47	0.00051	1.28
ILMN_1656196	E2F6	0.00014	1.32	0.00048	1.21
ILMN_1768101	HOXB6	0.00014	1.51	0.00007	1.57
ILMN_1767662	LASS6	0.00014	1.24	0.00008	1.26
ILMN_3248803	LOC729680	0.00014	1.51	0.00009	1.51
ILMN_1737426	PCMTD1	0.00014	1.39	0.00013	1.37
ILMN_1746368	SELT	0.00014	1.29	0.00006	1.34
ILMN_1655595	SERPINE2	0.00014	1.49	0.00284	1.20
ILMN_1838320	ONECUT2	0.00015	1.35	0.00051	1.24
ILMN_1703688	RASA2	0.00015	1.29	0.00196	1.15
ILMN_1708934	ADM	0.00018	1.19	0.00014	1.19
ILMN_1678998	LRRC14	0.00018	1.20	0.00325	1.09
ILMN_1691237	CAP2	0.00018	1.30	0.00044	1.22
ILMN_1754234	ZMYND11	0.00018	1.22	0.00007	1.26
ILMN_2186216	GOLPH4	0.00019	1.24	0.00127	1.14
ILMN_2086105	SPRY4	0.00019	1.24	0.00021	1.21
ILMN_2121408	HBEGF	0.00020	1.27	0.00330	1.13
ILMN_1655229	SLC7A11	0.00020	1.49	0.00164	1.28
ILMN_1761363	VAMP4	0.00020	1.22	0.00046	1.17
ILMN_1652797	FAM174B	0.00024	1.29	0.00031	1.26
ILMN_1682781	TEAD2	0.00024	1.16	0.00006	1.22
ILMN_3267670	LOC100130550	0.00025	1.15	0.00051	1.12
ILMN_1790891	CKAP4	0.00026	1.31	0.00016	1.33
ILMN_2311020	DNAJC12	0.00028	1.42	0.00042	1.35
ILMN_1689046	FLJ20273	0.00030	1.38	0.00016	1.43
ILMN_1717056	TXNRD1	0.00030	1.29	0.00124	1.20
ILMN_1723412	ASCL2	0.00033	1.27	0.00020	1.28
ILMN_1761086	VPS54	0.00036	1.27	0.00285	1.16
ILMN_1724148	ORAI1	0.00036	1.14	0.00021	1.15
ILMN_1735220	CAV2	0.00038	1.37	0.00029	1.37
ILMN_2185884	DHRS4	0.00038	1.16	0.00030	1.16
ILMN_1720303	OSTM1	0.00038	1.31	0.00018	1.35
ILMN_1667670	SLC25A15	0.00038	1.28	0.00011	1.36
ILMN_1714461	RNF14	0.00046	1.21	0.00331	1.13
ILMN_1728305	PUM2	0.00046	1.10	0.00007	1.14
ILMN_1772692	DICER1	0.00047	1.31	0.00006	1.53
ILMN_1742109	DNAJC19	0.00051	1.16	0.00038	1.16
ILMN_2096191	AASDHPPT	0.00052	1.58	0.00233	1.37
ILMN_1768719	RDH11	0.00055	1.22	0.00036	1.23
ILMN_2067607	TMEM106B	0.00055	1.28	0.00021	1.33
ILMN_1866887	HS.294103	0.00055	1.20	0.00331	1.12
ILMN_1727633	PVRL3	0.00059	1.20	0.00318	1.13
ILMN_2306955	ACPL2	0.00062	1.23	0.00006	1.40
ILMN_2398926	C17orf58	0.00062	1.28	0.00005	1.58

ILMN_2206098	ATG10	0.00063	1.44	0.00084	1.38
ILMN_1788689	PHIP	0.00063	1.35	0.00031	1.39
ILMN_1671314	UXT	0.00063	1.16	0.00075	1.15
ILMN_2054725	SUGT1	0.00064	1.15	0.00016	1.19
ILMN_1695853	CLK4	0.00068	1.24	0.00333	1.15
ILMN_1795228	ZFAND5	0.00068	1.17	0.00131	1.14
ILMN_1755954	CPEB3	0.00069	1.22	0.00006	1.40
ILMN_1766000	PM20D2	0.00071	1.23	0.00020	1.30
ILMN_1693227	ZC3H7A	0.00071	1.16	0.00020	1.20
ILMN_1663919	TFF2	0.00072	1.44	0.00031	1.51
ILMN_3231638	FAM160B1	0.00080	1.24	0.00007	1.43
ILMN_2352580	MBD1	0.00080	1.14	0.00036	1.15
ILMN_2155228	CRLF3	0.00082	1.37	0.00022	1.47
ILMN_1812618	ARAP3	0.00085	1.16	0.00180	1.13
ILMN_1768534	BHLHB2	0.00085	1.13	0.00051	1.14
ILMN_1700515	C17orf58	0.00085	1.26	0.00006	1.51
ILMN_1793743	DIRC2	0.00085	1.16	0.00035	1.19
ILMN_1658483	IL1A	0.00085	1.27	0.00108	1.24
ILMN_1663873	MMP13	0.00085	1.19	0.00171	1.15
ILMN_2319588	OSGIN1	0.00085	1.32	0.00142	1.27
ILMN_1663407	SURF1	0.00085	1.15	0.00058	1.16
ILMN_1786396	ZZEF1	0.00085	1.16	0.00184	1.12
ILMN_1785158	HERPUD2	0.00089	1.44	0.00044	1.50
ILMN_1710124	CMTM8	0.00090	1.20	0.00148	1.17
ILMN_3243471	CNPY2	0.00090	1.21	0.00233	1.16
ILMN_1722025	CPEB4	0.00090	1.24	0.00109	1.22
ILMN_1778523	KLF9	0.00090	1.27	0.00148	1.23
ILMN_1670079	OMA1	0.00090	1.16	0.00171	1.14
ILMN_2080637	ZBTB44	0.00090	1.14	0.00280	1.11
ILMN_3224126	LOC729222	0.00092	1.17	0.00228	1.13
ILMN_1730433	CD2AP	0.00094	1.31	0.00319	1.23
ILMN_1730928	CDK5R1	0.00094	1.29	0.00103	1.27
ILMN_1698395	GNB5	0.00098	1.19	0.00007	1.35
ILMN_2302654	LRP8	0.00098	1.27	0.00247	1.21
ILMN_1684034	STAT5B	0.00098	1.16	0.00085	1.16
ILMN_1732555	B4GALT6	0.00107	1.28	0.00070	1.30
ILMN_1700834	SLK	0.00115	1.15	0.00068	1.17
ILMN_2120695	TSPAN7	0.00115	1.33	0.00144	1.30
ILMN_1686871	PARP1	0.00116	1.21	0.00031	1.27
ILMN_1747183	GXYLT1	0.00117	1.30	0.00070	1.33
ILMN_1775192	BCLAF1	0.00118	1.14	0.00334	1.11
ILMN_1798270	C11orf75	0.00118	1.15	0.00011	1.26
ILMN_1699112	COPB1	0.00118	1.18	0.00329	1.13
ILMN_1756572	COQ2	0.00118	1.10	0.00009	1.18
ILMN_2378100	FBXL5	0.00118	1.27	0.00068	1.30
ILMN_3307901	GAN	0.00118	1.22	0.00330	1.17
ILMN_1809292	IMMP2L	0.00118	1.25	0.00119	1.24
ILMN_1739876	RAB3GAP1	0.00118	1.10	0.00115	1.10
ILMN_1726496	SEL1L	0.00118	1.22	0.00291	1.17
ILMN_1759792	CLIP4	0.00119	1.16	0.00007	1.33
ILMN_1729905	GAL3ST1	0.00119	1.31	0.00059	1.36
ILMN_1770663	KRT24	0.00119	1.12	0.00020	1.18
ILMN_2263144	MGC3196	0.00119	1.14	0.00035	1.18
ILMN_1652787	PIK3AP1	0.00119	1.23	0.00098	1.24
ILMN_1702683	SLC33A1	0.00119	1.17	0.00067	1.19
ILMN_2117904	ZNF22	0.00119	1.16	0.00147	1.15
ILMN_1801605	BIRC6	0.00120	1.14	0.00261	1.12
ILMN_2353033	FUBP3	0.00120	1.14	0.00011	1.24

ILMN_2364928	APBA2BP	0.00121	1.21	0.00042	1.26
ILMN_1759464	C1orf124	0.00121	1.09	0.00014	1.15
ILMN_1690546	PPP3CC	0.00121	1.11	0.00228	1.10
ILMN_1788540	LOC732075	0.00126	1.10	0.00014	1.16
ILMN_1687757	AKR1C4	0.00127	1.17	0.00048	1.21
ILMN_1813475	HERC2	0.00127	1.16	0.00057	1.19
ILMN_1761808	MCFD2	0.00129	1.25	0.00094	1.27
ILMN_2366972	NUDT6	0.00130	1.11	0.00002	1.32
ILMN_1741175	RAB11FIP2	0.00130	1.17	0.00148	1.17
ILMN_1906520	HS.417262	0.00130	1.26	0.00031	1.37
ILMN_3238358	SCARNA22	0.00131	1.16	0.00211	1.14
ILMN_1705111	FNDC3A	0.00133	1.35	0.00309	1.29
ILMN_1693891	MOGAT2	0.00136	1.14	0.00211	1.13
ILMN_1806667	FRAS1	0.00139	1.12	0.00246	1.11
ILMN_1769191	GNAS	0.00141	1.09	0.00144	1.10
ILMN_1665877	RNF149	0.00144	1.19	0.00068	1.23
ILMN_1660635	LACTB2	0.00149	1.21	0.00021	1.32
ILMN_1676197	LRP11	0.00158	1.19	0.00057	1.23
ILMN_1703015	ZRANB2	0.00158	1.18	0.00214	1.17
ILMN_2184612	C3orf52	0.00161	1.24	0.00070	1.29
ILMN_1696360	CTSB	0.00170	1.17	0.00040	1.23
ILMN_1694311	NUDT6	0.00177	1.17	0.00009	1.35
ILMN_1679693	TMEM87B	0.00178	1.30	0.00229	1.29
ILMN_1666269	CTSZ	0.00181	1.15	0.00020	1.25
ILMN_1814213	PQLC3	0.00187	1.17	0.00044	1.23
ILMN_1750092	SEPSECS	0.00187	1.15	0.00195	1.15
ILMN_2255605	TULP4	0.00192	1.17	0.00233	1.16
ILMN_2062271	CDK5R1	0.00195	1.22	0.00148	1.23
ILMN_1704446	SLC6A10P	0.00195	1.25	0.00148	1.26
ILMN_1688381	BPTF	0.00197	1.08	0.00071	1.10
ILMN_2225144	EIF4E3	0.00197	1.17	0.00233	1.17
ILMN_2143685	CLDN7	0.00199	1.11	0.00007	1.24
ILMN_1793932	CRB3	0.00199	1.18	0.00148	1.19
ILMN_1788625	EXOC5	0.00199	1.21	0.00144	1.22
ILMN_1658989	MEX3B	0.00208	1.15	0.00214	1.15
ILMN_3228822	TMEM194A	0.00216	1.17	0.00021	1.30
ILMN_1733311	PIGB	0.00221	1.20	0.00071	1.26
ILMN_1766981	UNC50	0.00232	1.22	0.00107	1.26
ILMN_1792587	VPS4B	0.00232	1.18	0.00035	1.28
ILMN_1704196	DSG2	0.00236	1.16	0.00196	1.17
ILMN_2371911	MUC1	0.00241	1.10	0.00073	1.13
ILMN_1734198	RNGTT	0.00241	1.26	0.00307	1.24
ILMN_1697642	BCAP29	0.00241	1.07	0.00148	1.08
ILMN_1693401	KLHL28	0.00242	1.21	0.00033	1.34
ILMN_1682919	PAFAH2	0.00243	1.13	0.00090	1.17
ILMN_1731353	CHPF	0.00246	1.30	0.00310	1.28
ILMN_1749834	LOC388588	0.00259	1.15	0.00259	1.15
ILMN_3251686	UBTD2	0.00259	1.16	0.00090	1.20
ILMN_1777318	C9orf64	0.00273	1.16	0.00044	1.25
ILMN_1660436	HSPA1B	0.00274	1.13	0.00094	1.16
ILMN_1704972	TRIM5	0.00274	1.21	0.00055	1.30
ILMN_1666609	USP22	0.00274	1.12	0.00325	1.12
ILMN_1661189	ZBTB41	0.00274	1.11	0.00242	1.11
ILMN_1743204	DUSP8	0.00275	1.19	0.00331	1.18
ILMN_1862217	HS.532698	0.00283	1.17	0.00285	1.17
ILMN_1707503	C1orf144	0.00286	1.14	0.00212	1.15
ILMN_1744830	ARHGAP11A	0.00299	1.06	0.00002	1.21
ILMN_2405602	OSBPL1A	0.00299	1.06	0.00021	1.10

ILMN_1740426	RASD1	0.00299	1.13	0.00264	1.13
ILMN_2395204	SLTM	0.00317	1.13	0.00271	1.13
ILMN_1687084	C3orf64	0.00320	1.18	0.00229	1.20
ILMN_1670000	DCAF6	0.00320	1.06	0.00322	1.06
ILMN_2404085	CLIP1	0.00322	1.16	0.00040	1.26
ILMN_1864422	HS.283402	0.00322	1.17	0.00212	1.19
ILMN_3300353	LOC729920	0.00327	1.06	0.00146	1.08
ILMN_2366246	SEC23B	0.00327	1.11	0.00334	1.11
ILMN_1809496	COPG2	0.00330	1.10	0.00014	1.20
ILMN_2225608	FAM135A	0.00330	1.18	0.00031	1.31
ILMN_1732985	PHF20L1	0.00330	1.12	0.00051	1.18
ILMN_1805778	RBM12B	0.00330	1.21	0.00115	1.27

* False discovery rate (FDR) adjusted p-value
