# Supplementary material for: LRH-1 drives colon cancer cell growth by repressing the expression of the CDKN1A gene in a p53-dependent manner
Source: Nucleic Acids Res. 2015 Sep 22;44(2):582–94. doi: 10.1093/nar/gkv948 (PMC4737183; doi:10.1093/nar/gkv948)
Supplement: SUPPLEMENTARY DATA [file supp_gkv948_nar-01236-x-2015-File019.pdf]

Supplementary Table 6. Genes down-regulated in HT29 cells following LRH-1 siRNA

		HT29 siLRH-1 #1 vs siLuc		HT29 siLRH-1 #2 vs siLuc	
Probeset ID	*Gene Symbol*	*p-value**	*Fold Change*	*p-value**	*Fold Change*
ILMN_1692267	B4GALT3	0.00003	-1.26	0.00001	-1.32
ILMN_3176090	LOC100130919	0.00003	-1.11	0.00000	-1.37
ILMN_1681304	PAN3	0.00003	-1.51	0.00004	-1.40
ILMN_1697959	SLC35B4	0.00003	-1.51	0.00043	-1.25
ILMN_1804277	SPRED1	0.00003	-1.32	0.00033	-1.17
ILMN_1798957	C12orf47	0.00005	-1.17	0.00272	-1.06
ILMN_2098616	C5orf39	0.00007	-1.46	0.00159	-1.20
ILMN_3217625	LOC100132402	0.00009	-1.09	0.00002	-1.11
ILMN_1721842	RYBP	0.00009	-1.24	0.00005	-1.23
ILMN_2336186	LCMT1	0.00010	-1.19	0.00050	-1.12
ILMN_1792689	HIST1H2AC	0.00011	-1.33	0.00060	-1.21
ILMN_1747204	HTRA2	0.00011	-1.39	0.00192	-1.18
ILMN_1785286	SOCS5	0.00011	-1.21	0.00006	-1.20
ILMN_2415011	DCTD	0.00014	-1.26	0.00044	-1.18
ILMN_1782069	TRAK1	0.00014	-1.43	0.00182	-1.21
ILMN_1669273	PPT1	0.00016	-1.31	0.00033	-1.25
ILMN_1669703	TNK2	0.00016	-1.19	0.00043	-1.15
ILMN_2104696	ERICH1	0.00018	-1.22	0.00031	-1.18
ILMN_1707337	MSTO1	0.00024	-1.40	0.00073	-1.28
ILMN_1685742	NIPA2	0.00024	-1.47	0.00135	-1.29
ILMN_1796669	PSEN1	0.00024	-1.14	0.00004	-1.17
ILMN_3251085	RBBP4	0.00024	-1.49	0.00159	-1.29
ILMN_2128770	CDR2L	0.00024	-1.20	0.00051	-1.16
ILMN_3236713	SNHG1	0.00024	-1.16	0.00004	-1.22
ILMN_2304495	PPP1R1B	0.00028	-1.28	0.00011	-1.31
ILMN_1768110	ZAK	0.00028	-1.18	0.00120	-1.13
ILMN_1733396	CDC25A	0.00028	-1.21	0.00032	-1.20
ILMN_2393450	C14orf173	0.00029	-1.11	0.00009	-1.13
ILMN_1723116	AMFR	0.00032	-1.26	0.00001	-1.53
ILMN_2157075	LRCH4	0.00032	-1.39	0.00288	-1.22
ILMN_1763162	DPH2	0.00036	-1.20	0.00043	-1.18
ILMN_1721729	PPARBP	0.00036	-1.25	0.00045	-1.23
ILMN_1757347	C22orf9	0.00039	-1.16	0.00072	-1.13
ILMN_1765060	FBXO34	0.00039	-1.17	0.00202	-1.11
ILMN_2073446	C1orf116	0.00039	-1.37	0.00026	-1.41
ILMN_1692271	CCM2	0.00039	-1.29	0.00205	-1.19
ILMN_1764127	NPHP1	0.00039	-1.21	0.00308	-1.13
ILMN_1652445	RAC1	0.00039	-1.23	0.00032	-1.23
ILMN_1758613	RAPGEFL1	0.00039	-1.25	0.00004	-1.38
ILMN_1706553	SMG7	0.00040	-1.20	0.00336	-1.12
ILMN_1770084	TACC1	0.00040	-1.31	0.00154	-1.22
ILMN_1744574	TRUB1	0.00040	-1.38	0.00176	-1.26
ILMN_2096719	GRK5	0.00044	-1.23	0.00043	-1.23
ILMN_1722292	AVL9	0.00046	-1.25	0.00154	-1.18
ILMN_1774708	ORMDL2	0.00046	-1.27	0.00136	-1.20
ILMN_1708093	ARHGEF5	0.00052	-1.12	0.00002	-1.21
ILMN_1657429	SMAGP	0.00053	-1.23	0.00115	-1.19
ILMN_2383435	PSMD10	0.00060	-1.34	0.00100	-1.29
ILMN_1775330	C15orf52	0.00063	-1.34	0.00028	-1.41
ILMN_1723706	GRAMD4	0.00063	-1.23	0.00137	-1.19
ILMN_1738589	MGLL	0.00063	-1.30	0.00103	-1.26
ILMN_1673543	PGM2	0.00063	-1.13	0.00281	-1.09
ILMN_1800425	SLC9A1	0.00063	-1.24	0.00000	-2.39
ILMN_1727589	SULT2B1	0.00063	-1.23	0.00021	-1.29

ILMN_1755737	TRABD	0.00063	-1.12	0.00001	-1.29
ILMN_2130635	FOXRED2	0.00064	-1.17	0.00132	-1.15
ILMN_3293843	LOC100132948	0.00065	-1.24	0.00031	-1.29
ILMN_1761044	GNB1L	0.00066	-1.15	0.00063	-1.15
ILMN_1735955	LOC644033	0.00069	-1.23	0.00176	-1.18
ILMN_1705814	KRT80	0.00076	-1.35	0.00001	-2.11
ILMN_1795285	PHF15	0.00080	-1.16	0.00072	-1.16
ILMN_1769201	ELF3	0.00087	-1.17	0.00139	-1.15
ILMN_3178307	KRT18P28	0.00087	-1.12	0.00132	-1.11
ILMN_1752837	ARL8B	0.00093	-1.11	0.00005	-1.18
ILMN_2413158	PODXL	0.00093	-1.19	0.00182	-1.16
ILMN_3244114	DHRS11	0.00093	-1.12	0.00072	-1.13
ILMN_1703852	EFNB2	0.00093	-1.25	0.00008	-1.42
ILMN_1718734	MLLT6	0.00093	-1.24	0.00042	-1.29
ILMN_1678805	POMT2	0.00093	-1.19	0.00073	-1.19
ILMN_1728228	ZNF548	0.00093	-1.14	0.00072	-1.14
ILMN_1770048	DPH5	0.00097	-1.17	0.00004	-1.34
ILMN_2081682	SMAP2	0.00097	-1.23	0.00073	-1.24
ILMN_2226955	VOPP1	0.00097	-1.21	0.00002	-1.48
ILMN_1771629	C14orf124	0.00106	-1.10	0.00001	-1.30
ILMN_1729033	RPL9	0.00106	-1.12	0.00002	-1.26
ILMN_1784367	HSPD1	0.00125	-1.15	0.00039	-1.20
ILMN_1723625	MAP4K2	0.00125	-1.10	0.00001	-1.28
ILMN_3233205	SNHG7	0.00125	-1.13	0.00073	-1.14
ILMN_3256325	CYB561D1	0.00139	-1.19	0.00008	-1.35
ILMN_2316104	IQCB1	0.00139	-1.24	0.00132	-1.24
ILMN_2137084	LIN9	0.00139	-1.14	0.00103	-1.15
ILMN_3300663	LOC728661	0.00139	-1.16	0.00001	-1.46
ILMN_1753683	SOCS7	0.00139	-1.22	0.00009	-1.38
ILMN_1750160	FASTKD3	0.00140	-1.18	0.00190	-1.17
ILMN_3235397	SNORD110	0.00140	-1.09	0.00029	-1.13
ILMN_2199284	ANAPC7	0.00143	-1.11	0.00202	-1.10
ILMN_1664630	CHEK1	0.00144	-1.17	0.00069	-1.20
ILMN_1674151	GCOM1	0.00158	-1.17	0.00072	-1.21
ILMN_3294033	LOC339970	0.00158	-1.18	0.00288	-1.15
ILMN_2216918	SHPK	0.00158	-1.09	0.00104	-1.10
ILMN_1752639	SLC25A24	0.00163	-1.21	0.00050	-1.28
ILMN_1851107	HS.280924	0.00171	-1.11	0.00197	-1.10
ILMN_1663447	HNRNPA1	0.00174	-1.26	0.00280	-1.23
ILMN_1735402	WDR22	0.00174	-1.16	0.00050	-1.21
ILMN_1679617	LOC654155	0.00178	-1.20	0.00115	-1.23
ILMN_1706483	C1orf116	0.00186	-1.19	0.00001	-1.57
ILMN_1676980	MTSS1	0.00186	-1.17	0.00135	-1.19
ILMN_1657128	PTPRU	0.00193	-1.36	0.00120	-1.41
ILMN_1906541	HS.232520	0.00200	-1.16	0.00253	-1.15
ILMN_2060413	CD24	0.00201	-1.17	0.00010	-1.31
ILMN_1782141	GRHL3	0.00201	-1.21	0.00176	-1.22
ILMN_1798657	TBL1XR1	0.00208	-1.30	0.00308	-1.27
ILMN_1764851	TP53RK	0.00217	-1.19	0.00002	-1.51
ILMN_2412024	JMJD6	0.00217	-1.21	0.00288	-1.19
ILMN_1657708	MGLL	0.00217	-1.18	0.00202	-1.18
ILMN_1700345	RNF41	0.00217	-1.12	0.00033	-1.19
ILMN_1732071	HIST2H2BE	0.00235	-1.12	0.00063	-1.16
ILMN_1725306	RNF6	0.00254	-1.19	0.00283	-1.18
ILMN_1795856	LOC644935	0.00262	-1.17	0.00336	-1.16
ILMN_2205695	C4orf28	0.00282	-1.20	0.00176	-1.22
ILMN_1752988	C11orf17	0.00286	-1.14	0.00008	-1.29
ILMN_3235404	SNORA57	0.00299	-1.15	0.00013	-1.30

ILMN_1763520	SULT2B1	0.00299	-1.27	0.00132	-1.33
ILMN_2366445	KRT80	0.00306	-1.22	0.00005	-1.56
ILMN_2281128	PSMD10	0.00310	-1.16	0.00056	-1.23
ILMN_1792825	ARIH2	0.00314	-1.18	0.00202	-1.20
ILMN_1694588	C4BPB	0.00315	-1.14	0.00004	-1.36
ILMN_1695420	CLTA	0.00315	-1.18	0.00281	-1.18
ILMN_1774722	CGREF1	0.00320	-1.12	0.00084	-1.16
ILMN_1796179	HIST1H2BK	0.00344	-1.10	0.00042	-1.16
ILMN_1703180	ETV3	0.00347	-1.11	0.00044	-1.18
ILMN_2394242	AMMECR1	0.00347	-1.14	0.00277	-1.15
ILMN_1670959	CEACAM5	0.00348	-1.16	0.00330	-1.16
ILMN_1775823	POFUT2	0.00348	-1.08	0.00247	-1.09

* False discovery rate (FDR) adjusted p-value
