# Supplementary material for: LRH-1 drives colon cancer cell growth by repressing the expression of the CDKN1A gene in a p53-dependent manner
Source: Nucleic Acids Res. 2015 Sep 22;44(2):582–94. doi: 10.1093/nar/gkv948 (PMC4737183; doi:10.1093/nar/gkv948)
Supplement: SUPPLEMENTARY DATA [file supp_gkv948_nar-01236-x-2015-File020.pdf]

Supplementary Table 7. GO analysis of LRH-1 regulated genes using DAVID
HCT116 unique genes

Term		*p-value*	*Fold Enrichment*
GO:0051726	regulation of cell cycle	1.64E-05	2.80
GO:0007049	cell cycle	4.58E-04	1.84
GO:0015031	protein transport	6.57E-04	1.83
GO:0008104	protein localization	6.90E-04	1.75
GO:0033554	cellular response to stress	7.59E-04	1.98
GO:0045184	establishment of protein localization	7.83E-04	1.81
GO:0006259	DNA metabolic process	1.46E-03	1.99
GO:0022402	cell cycle process	1.55E-03	1.92
GO:0042981	regulation of apoptosis	1.69E-03	1.73
GO:0010033	response to organic substance	1.93E-03	1.77
GO:0009719	response to endogenous stimulus	1.95E-03	2.10
GO:0043067	regulation of programmed cell death	1.98E-03	1.71
GO:0010941	regulation of cell death	2.12E-03	1.71
GO:0043066	negative regulation of apoptosis	2.14E-03	2.18
GO:0006282	regulation of DNA repair	2.20E-03	8.78
GO:0043069	negative regulation of programmed cell death	2.50E-03	2.15
GO:0060548	negative regulation of cell death	2.55E-03	2.15
GO:0010604	positive regulation of macromolecule metabolic process	2.65E-03	1.67
GO:0070647	protein modification by small protein conjugation or removal	2.92E-03	2.90
GO:0007005	mitochondrion organization	3.06E-03	3.08
GO:0009725	response to hormone stimulus	3.13E-03	2.11
GO:0009057	macromolecule catabolic process	3.65E-03	1.68
GO:0051276	chromosome organization	3.73E-03	1.91
GO:0019941	modification-dependent protein catabolic process	3.90E-03	1.82
GO:0043632	modification-dependent macromolecule catabolic process	3.90E-03	1.82
GO:0044265	cellular macromolecule catabolic process	4.06E-03	1.71
GO:0016568	chromatin modification	5.08E-03	2.26
GO:0080135	regulation of cellular response to stress	5.14E-03	3.38
GO:0022403	cell cycle phase	5.41E-03	1.96
GO:0045739	positive regulation of DNA repair	6.15E-03	10.31
GO:0051603	proteolysis involved in cellular protein catabolic process	6.91E-03	1.74
GO:0032446	protein modification by small protein conjugation	7.20E-03	2.93
GO:0006916	anti-apoptosis	7.28E-03	2.44
GO:0044257	cellular protein catabolic process	7.31E-03	1.73
GO:0040008	regulation of growth	7.44E-03	2.04
GO:0000278	mitotic cell cycle	7.74E-03	1.98
GO:0032583	regulation of gene-specific transcription	7.92E-03	2.88
GO:0051173	positive regulation of nitrogen compound metabolic process	9.08E-03	1.68
GO:0006325	chromatin organization	9.42E-03	1.94
GO:0007242	intracellular signaling cascade	9.48E-03	1.45

HT29 unique genes

Term		p-value	Fold Enrichment
GO:0044265	cellular macromolecule catabolic process	7.99E-07	2.47
GO:0030163	protein catabolic process	1.62E-06	2.57
GO:0009057	macromolecule catabolic process	1.71E-06	2.36
GO:0051603	proteolysis involved in cellular protein catabolic process	2.19E-06	2.59
GO:0044257	cellular protein catabolic process	2.42E-06	2.57
GO:0043632	modification-dependent macromolecule catabolic process	7.30E-06	2.53
GO:0019941	modification-dependent protein catabolic process	7.30E-06	2.53
GO:0016192	vesicle-mediated transport	2.16E-05	2.44
GO:0015031	protein transport	4.03E-05	2.16
GO:0045184	establishment of protein localization	4.88E-05	2.14
GO:0006508	proteolysis	1.13E-04	1.89
GO:0008104	protein localization	2.91E-04	1.92
GO:0032446	protein modification by small protein conjugation	3.94E-04	4.04
GO:0046907	intracellular transport	4.81E-04	2.07
GO:0070647	protein modification by small protein conjugation or removal	1.74E-03	3.33
GO:0016567	protein ubiquitination	3.16E-03	3.67
GO:0006986	response to unfolded protein	3.34E-03	4.78
GO:0006886	intracellular protein transport	4.64E-03	2.20
GO:0034976	response to endoplasmic reticulum stress	4.98E-03	7.13
GO:0034613	cellular protein localization	4.99E-03	2.12
GO:0043161	proteasomal ubiquitin-dependent protein catabolic process	5.04E-03	3.80
GO:0010498	proteasomal protein catabolic process	5.04E-03	3.80
GO:0070727	cellular macromolecule localization	5.32E-03	2.11
GO:0006984	ER-nuclear signaling pathway	5.54E-03	6.93
GO:0035116	embryonic hindlimb morphogenesis	5.57E-03	10.77
GO:0010033	response to organic substance	7.38E-03	1.75
GO:0007005	mitochondrion organization	7.67E-03	3.16
GO:0051726	regulation of cell cycle	8.70E-03	2.20

HCT116 and HT29 Shared Genes

Term		p-value	Fold Enrichment
GO:0034976	response to endoplasmic reticulum stress	1.33E-03	18.09
GO:0006984	ER-nucleus signaling pathway	1.45E-03	17.57
GO:0051130	positive regulation of cellular component organization	6.19E-03	5.10
GO:0015031	protein transport	9.46E-03	2.42
